# Supplementary material for: Grafting of Anionic Decahydro-Closo-Decaborate Clusters on Keggin and Dawson-Type Polyoxometalates: Syntheses, Studies in Solution, DFT Calculations and Electrochemical Properties
Source: Molecules. 2022 Nov 8;27(22):7663. doi: 10.3390/molecules27227663 (PMC9694426; doi:10.3390/molecules27227663)
Supplement: Supplementary file 1 [file molecules-27-07663-s001.zip › molecules-1977327-supplementary.pdf]

# *Supporting Information*

## Grafting of anionic decahydro-*closo*-decaborate clusters on Keggin and Dawson-type polyoxometalates. Syntheses, studies in solution, DFT calculations and electrochemical properties

Manal Diab,[a,b] Ana Mateo,[c] Joumada El Cheikh,[d] Zeinab El Hajj,[a,b] Mohamed Haouas,[a] Alireza Ranjbari,[d,e] Vincent Guérineau,[f] David Touboul,[f] Nathalie Leclerc,[a] Emmanuel Cadot,[a] Daoud Naoufal,[b,*] Carles Bo,[c,*] and Sébastien Floquet,[a,*]

a. Institut Lavoisier de Versailles, CNRS, UVSQ, Université Paris-Saclay, 45 av. des Etats-Unis, 78035 Versailles, France
b. Laboratory of Organometallic and coordination chemistry, LCIO, Lebanese University, Faculty of Sciences I, Hadath, Lebanon
c. Institute of Chemical Research of Catalonia (ICIQ). The Barcelona Institute of Science and Technology. Av. Països Catalans 16, 43007 Tarragona, Spain
d. Equipe de Recherche et Innovation en Electrochimie pour l'énergie (ERIEE), Institut de Chimie Moléculaire et des matériaux d'Orsay (ICMMO), UMR CNRS 8182, Université Paris-Sud, Université Paris-Saclay, 91405 Orsay, France
e. Université Paris-Saclay, CNRS, Institut de Chimie Physique, UMR 8000, 91405 Orsay, France
f. Institut de Chimie des Substances Naturelles, CNRS UPR2301, Université Paris-Sud, Université Paris-Saclay, Avenue de la Terrasse, 91198 Gif-sur-Yvette Cedex, France

***Corresponding authors:** sebastien.floquet@uvsq.fr, cbo@iciq.cat, dnaoufal@ul.edu.lb

*Copyrights : Figures S1-S12, S17, S19-S22, S24-S28, and S30-S41 are reproduced with permission from the doctoral thesis manuscript of Dr Manal Diab, University Paris Saclay/Lebanese University, May 2018.*

## Table of content


### 1. Experimental section

1.1 General methods

1.2 Optimization of syntheses thanks to NMR titration studies

1.2.1 $SiW_{10}$ derivatives

1.2.2 $P_2W_{17}$ derivatives

1.3 Syntheses of products

## 2. Characterization of adducts

2.1 FT-IR spectra

2.2 MALDI-TOF data and spectra

2.3 NMR characterizations

## 3. DFT calculation

Computational details and additional figures

## 4. Electronic and electrochemical studies.

4.1 Electronic spectra

4.2 Electrochemical properties

4.3 Electrocatalytic properties

# Part 1: Experimental section

## 1-1 General methods

**Fourier Transform Infrared (FT-IR)** spectra were recorded on a 6700 FT-IR Nicolet spectrophotometer, using diamond ATR technique. The spectra were recorded on non-diluted compounds and ATR correction was applied. **Elemental analyses** of C, H, and N were carried out by the analytical service of the CNRS at Gif sur Yvette, France. Quantitative analyses of metals were carried out by ICP analysis, performed in CREALINS laboratory in Vernaison, France. **Water contents** were determined by thermal gravimetric analysis (TGA) with a Mettler Toledo TGA/DSC 1, STAR$^{e}$ System apparatus under oxygen or nitrogen flow (50 mL min$^{-1}$) at a heating rate of 5 °C min$^{-1}$ up to 700 °C. **UV-vis spectra** were recorded on a Perkin-Elmer UV-vis-NIR Lambda-750 spectrometer using calibrated 0.1 cm Quartz-cell.

**Nuclear magnetic resonance (NMR)** solution spectra were recorded at 25 °C. $^{1}$H, $^{11}$B, $^{13}$C, $^{15}$N, $^{29}$Si, and $^{31}$P NMR were measured with a Bruker Avance 400 MHz spectrometer equipped with a 5 mm BBI probe head and operated at a magnetic field strength of 9.4 T. The $^{183}$W NMR spectra were measured on a Bruker Avance 500 spectrometer at a resonance frequency of 20.8 MHz equipped with a specific low-gamma nuclei 10 mm probe head. Quartz NMR tubes are used to avoid background signals from the standard glass tubes in case of $^{11}$B and $^{29}$Si. $CD_3CN$ was used as the solvent. Typically, $^{1}$H spectra were recorded with one pulse sequence at 30° flip angle (pulse duration 2.4 -µs), using 1 s recycle delay, 1.6 s acquisition time, and 80 number of scans. 2D $^{1}$H-$^{1}$H ROESY spectra were carried out on some selected samples using standard phase sensitive pulse sequences in States mode and 300 ms mixing time. The COSY spectra were recorded by using 1 s recycle delay. To record F2 dimension, 5 kHz spectral width, 0.8 s acquisition time, eight scans, and 8192 data points were employed, whereas 5 kHz spectral width and 956 t1 increments were used to record F1 dimension. In case of samples containing boron, Waltz16 $^{11}$B decoupling was applied during the acquisition of $^{1}$H spectra. The $^{11}$B spectra were recorded with Hahn echo sequence (echo delay 117 -µs) under proton decoupling condition, using 0.1 s recycle delay, 21 ms acquisition time, and 1024 number of scans. The $^{13}$C spectra were obtained with either standard power-gated decoupling or Dept145 pulse sequences, using typically 4.5 s recycle delay, 1.3 s acquisition time, and ca. 8000 number of scans. In the $^{1}$H{$^{13}$C} HMBC experiments, the proton dimension was acquired by using 4096 data points, 5000 Hz spectral width, 64 scans, and 1 s recycle delay. The $^{13}$C dimension was recorded by using 254 t1 increments, and 25 kHz spectral width. To collect $^{1}$H{$^{15}$N} HMBC spectra of the solutions, the spectrometer was operated at 40.55 MHz for $^{15}$N. The spectral width for the nitrogen dimension was 20 kHz, and 64 t1 increments were used. For the proton dimension, the acquisition time and the spectral width were 0.4 s and 5000 Hz, respectively. The spectra were acquired with 512 scans and 1 s recycle delay. The $^{29}$Si NMR spectra were obtained with Hahn echo experiments under proton decoupling, a recycle delay of 6 s, and acquisition of around 8000 pulse transients. The $^{31}$P NMR spectra were run with 7.7 µs pulse duration (45° flip angle), 15 s recycle delay, 1 s acquisition time, and an

accumulation of 32 transients. For $^{183}W$ experiments, FIDs were accumulated with 90° pulses, a recycle time of 3 s, and a total number of FIDs of ca. 128000. Chemical shifts are reported relative to 1% $Me_4Si$ in $CDCl_3$ ($^{1}H$, $^{13}C$, and $^{29}Si$), nitromethane ($^{15}N$), 15% $BF_3.Et_2O$ in $CDCl_3$ ($^{11}B$), 85% $H_3PO_4$ ($^{31}P$), and 1 M $Na_2WO_4$ in $D_2O$ ($^{183}W$), according to conventional standards.[1]

**MALDI-TOF mass Spectrometry.** MALDI-TOF MS analyses were performed using an UltrafleXtreme mass spectrometer (Bruker Daltonics, Bremen). Acquisitions were performed in reflector or linear positive ion mode. The laser intensity was set just above the ion generation threshold to obtain peaks with the highest possible signal-to-noise (S/N) ratio without significant peak broadening. The mass spectrometer was externally calibrated using PEG1500 and PEG4500. All data were processed using the program FlexAnalysis (Bruker Daltonics, Bremen). Trans-2-[3-(4-ter-Butylphenyl)-2-propenylidene] malonitrile (DCTB, used as the matrix, the highest grade available and used without further purification) was purchased from Sigma Aldrich Co. Samples were prepared at a concentration of 60 µM in acetonitrile. The matrix solution was prepared at a concentration of 6 mM in THF. The sample was prepared by mixing the sample solution with matrix solution at a volume ratio of 1:9. After drying, the residues were analyzed by MALDI-TOF technique. Simulations of spectra were performed with IsoPro 3.1. Isopro is available as freeware. (https://sites.google.com/site/isoproms/home)

**Electrochemistry.** Cyclic voltammetry (CV) experiments were carried out with a PZG402 Potentiostat associated with an electrochemical analysis system (voltmaster 4). Prior, the chemical stability of the compounds was assessed by UV-Vis spectroscopy in $CH_3CN$ containing 0.1 M $TBAClO_4$, a medium in which the electrochemical studies will be performed. As far as absorbance, peak locations, and intensities are concerned, the obtained spectra remain identical for several days even in presence of an excess of acetic acid (20 eq/compound), a feature that suggests **$SiW_{10}$-APTES**, **$SiW_{10}$-mono$B_{10}$**, **$SiW_{10}$-di$B_{10}$**, **$P_2W_{17}$-APTES**, **$P_2W_{17}$-di$B_{10}$** and $[B_{10}H_9CO]^-$ are stable in the experimental conditions required for electrochemistry. Measurements were performed at room temperature in a conventional compartment cell with 3 electrodes and purge of argon gaz. A glassy carbon (GC) electrode with 0.07 $cm^2$ surface polished before the measurements was used as the working electrode. Potentials are measured against a saturated calomel reference electrode (SCE). The counter electrode was platinum of large surface area. Freshly distilled acetonitrile (ACN) was used throughout. The solutions were deaerated thoroughly for at least 45 minutes with pure argon and kept under a positive pressure of this gas during the experiments. The supporting electrolyte was 0.1 M Tetrabutylammonium perchlorate (TBAP) in $CH_3CN$. The electrocatalytic reduction of protons into hydrogen was evidenced by Gas Chromatography. A degassed $CH_3CN$ solution containing 0.2 mM of $P_2W_{17}$-APTES or $P_2W_{17}$-di$B_{10}$ in 0.1 M TBAP and $CH_3COOH$ (4 mM) was electrolyzed at 1.5 V vs SCE for 4 hours. During these controlled-potential Coulometry experiments, the cell was flushed with argon and the output gas was sampled (250 µL) every 42 min and analyzed using a PerkinElmer Clarus 850 gas chromatograph equipped with PoraPlot U columns thermostatted at 60 °C, and

MS5A columns thermostatted at 140 °C and TCD detector thermostatted at 160 °C and FID detector thermostatted at 180 °C.

## 1.2 Optimization of syntheses thanks to NMR titration studies

### 1.2.1 General procedure for NMR titration studies

$^{29}Si$, $^{31}P$ and $^{1}H$ NMR titrations studies were conducted on different mixtures $[B_{10}H_9CO]^-$/POM-APTES/DIPEA by varying the ratios of the three reactants to get the optimum conditions for the synthesis of the POM-$B_{10}$ adducts.

A constant quantity of $TBA_3H[(\gamma\text{-}SiW_{10}O_{36})(NH_2(CH_2)_3Si)_2O].3H_2O$, $SiW_{10}$-APTES, (0.25 g, 0.073 mmol) was dissolved in 0.5 mL of $CD_3CN$. TBA$[B_{10}H_9CO]$ in various amounts was then added and stirred till total solubility. The coupling reaction needs the presence of a base to help the deprotonation of the amine. A moderate and a bulky organic base, diisopropylethylamine (DIPEA), was used. For the titration experiments, liquid DIPEA was added drop by drop under stirring to the POM-APTES/$B_{10}H_9CO$ mixture. The solution was kept at room temperature in a closed flask for 3 hours, and then NMR spectra were measured ($^{1}H$, $^{29}Si$, $^{31}P$).

### 1.2.2 $SiW_{10}$ derivatives.

Several studies were done to assess i) the effect of the base and determine the optimal quantity of DIPEA needed and ii) the optimal ratio between all the reactants to get the target adducts.

For this purpose, we first fixed the ratio $B_{10}H_9CO/SiW_{10}$-APTES = 2 and we varied the ratio DIPEA/$SiW_{10}$-APTES from 0 to 4. The resulting $^{29}Si$ NMR spectra are shown in Figure S1. According to its $C_{2v}$ symmetry the $^{29}Si$ NMR spectrum of $SiW_{10}$-APTES precursor gives two signals at -62 ppm and -88 ppm, with 2:1 relative intensities assigned respectively to the APTES part and to the internal Si atom of the $SiW_{10}$ POM (see Figure S1a).[8-9] From $^{29}Si$ NMR spectra shown in Figure S1, it is obvious the base DIPEA plays a crucial role. Without addition of DIPEA, even with a $B_{10}H_9CO/SiW_{10}$-APTES ratio = 2 (Figure S1b), no reaction occurs and the spectrum can be superimposed to that of the precursor. For proportions $B_{10}H_9CO/SiW_{10}$-APTES/DIPEA = 2/1/2 (Figure S1c), two sets of signals assigned to two different species are observed. The first species is characterized by two signals at –61.6 and –63.4 ppm for the APTES part and one signal at –88.4 ppm for the Si center of the POM. The three signals display the same intensity and should characterize a species of lower symmetry compared to the precursor. This species is thus assigned to a mono adduct compound resulting from the grafting of one $[B_{10}H_9CO]^-$ cluster on one amine function of the APTES linker. Another minor species is characterized by two signals with intensities 2:1. A singlet at -62.4 ppm for the APTES part and a singlet at –88.6 ppm for the POM part. It differs significantly from the precursor and we assign these two peaks to a new species which could result from the grafting

of two $[B_{10}H_9CO]^-$ clusters noted as di-adduct. In this case, indeed, the apparent $C_{2V}$ symmetry must be recovered. Signals of the starting precursor are not seen. When the quantity of DIPEA increased the proportion of this di-adduct species increased concomitantly, whereas the signals of the mono-adduct decreases. Finally, for a proportions $B_{10}H_9CO$/$SiW_{10}$-APTES/DIPEA = 2/1/4, the di-adduct clearly dominates the spectrum (Figure S1e).

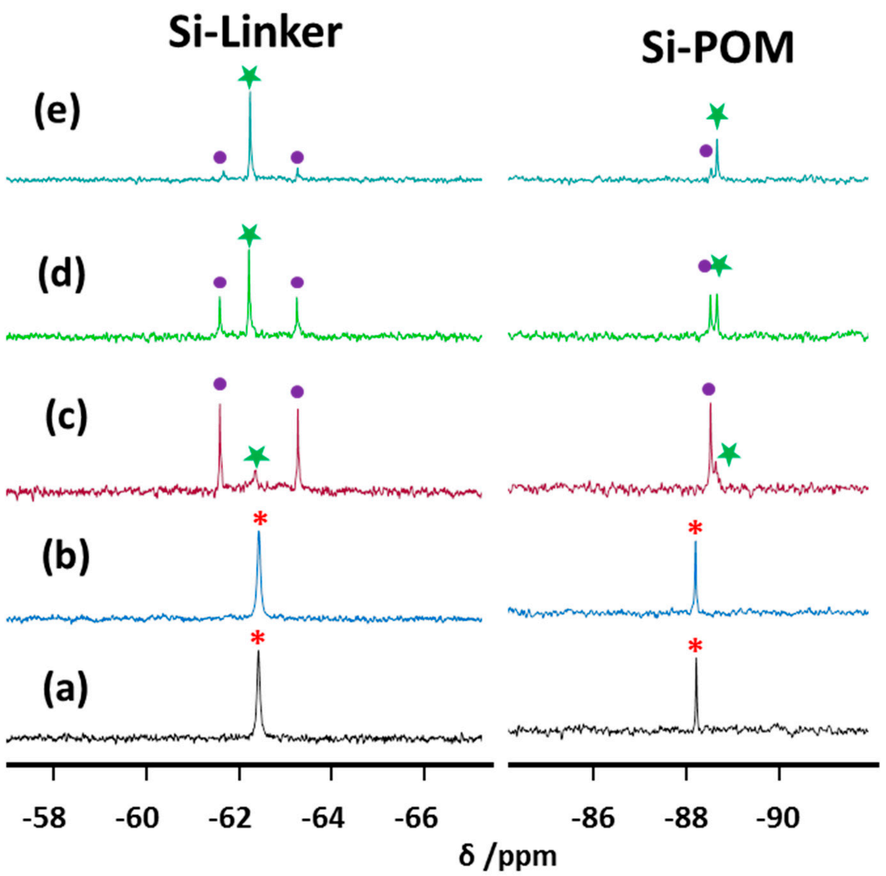


***Figure S1**: Effect of DIPEA amount shown in $^{29}$Si NMR spectra from the reaction medium in case of $SiW_{10}$-APTES with different Boron/$SiW_{10}$-APTES/DIPEA ratios .a) 0/1/0; b) 2/1/0; c) 2/1/2; d) 2/1/3; e) 2/1/4. Legend: * Unreacted $SiW_{10}$-APTES; ●mono-adduct; ✶ di-adduct*

Figure S2 represents the proportion of the three species in solution for ratio $B_{10}H_9CO$/$SiW_{10}$-APTES/DIPEA = 2/1/x, x ranging from 0 to 4. It evidences the crucial role of DIPEA in the reaction of $[B_{10}H_9CO]^-$ with $SiW_{10}$-APTES and demonstrates that it is possible to tune this reaction by playing on the amount of DIPEA. Two equivalents of base are needed for each $[B_{10}H_9CO]^-$ to be grafted to the $SiW_{10}$-APTES POM precursor.

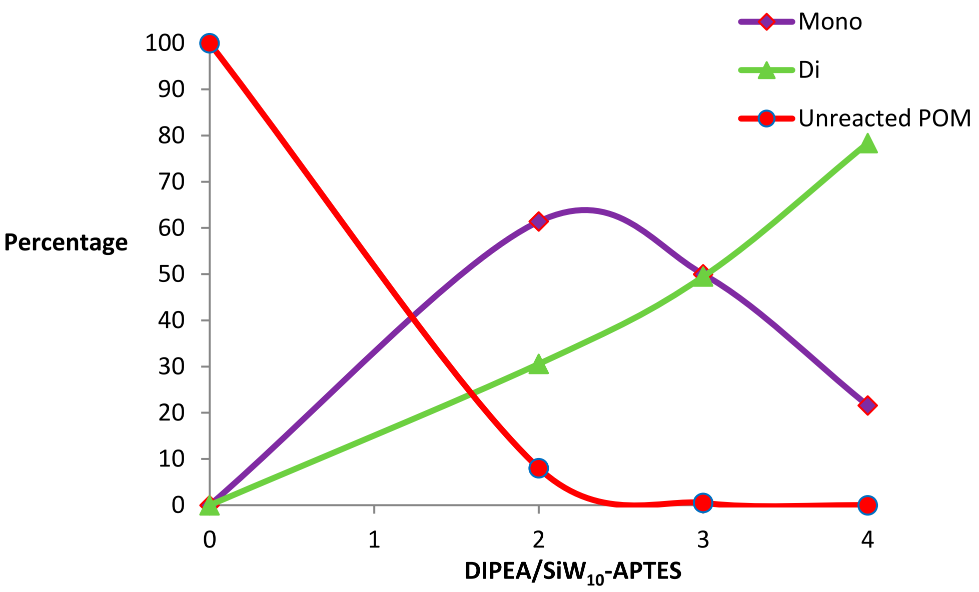


***Figure S2**: Graph showing the effect of DIPEA ratio on the formation of products in the $SiW_{10}$-APTES/$B_{10}H_9CO$/DIPEA system at fixed $B_{10}H_9CO$/$SiW_{10}$-APTES ratio = 2. The proportion of each species are determined by integration of the $^{29}$Si NMR signals*

Another set of experiments were done at a constant ratio DIPEA/ $B_{10}H_9CO$=2 with varying the ratio $B_{10}H_9CO$/$SiW_{10}$-APTES. The reaction was monitored by $^{29}$Si (Figure S3) and $^{1}$H NMR (Figure S4) for six different mixtures $SiW_{10}$-APTES/$B_{10}H_9CO$/DIPEA = 1/x/2x, x ranging from 0 to 3.

The resulting $^{29}$Si NMR spectra are given in Figure S3, each spectrum being recorded at least during one night to get a good signal/noise ratio. Starting from $SiW_{10}$-APTES, when a small proportion of $B_{10}H_9CO$ is added, three new small peaks of equal intensities appear at -61.6, -63.4 and -88.4 ppm, in agreement with the formation of mono-adduct already observed in the previous study. Concomitantly, the signals of the $SiW_{10}$-APTES precursor broaden and decrease until their disappearance for proportions above $SiW_{10}$-APTES/$B_{10}H_9CO$/DIPEA = 1/1/2. For this mixture the mono-adduct predominates while the di-adduct begins to be formed. The latter increases until becoming the single product for $SiW_{10}$-APTES/$B_{10}H_9CO$/DIPEA = 1/3/6, *i.e.* when an excess of $[B_{10}H_9CO]^-$ is added.

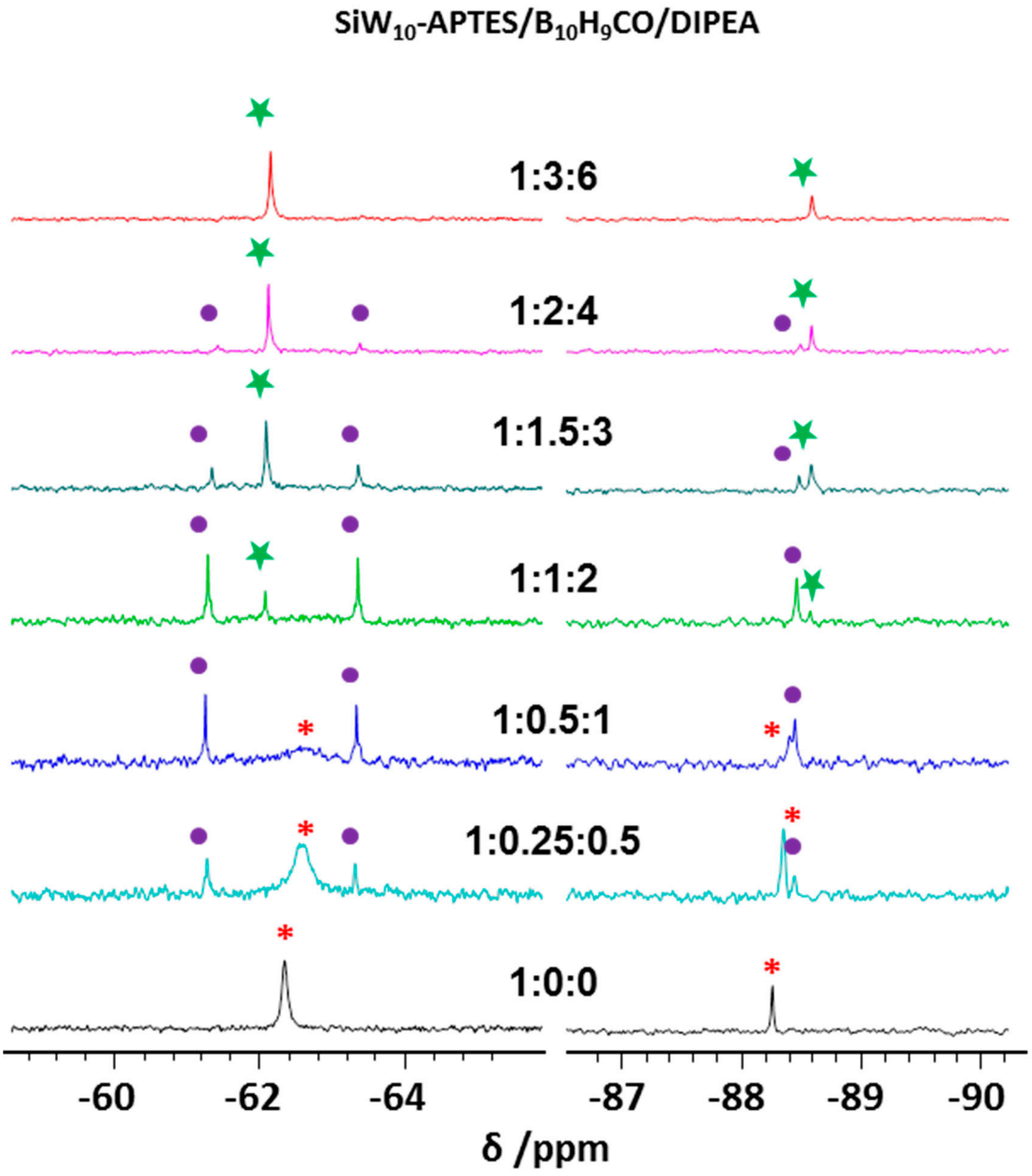


***Figure S3***: *$^{29}$Si NMR spectra obtained from the reaction medium with different $SiW_{10}$-APTES/$B_{10}H_9CO$/DIPEA ratios.*

*Legend: * Unreacted $SiW_{10}$-APTES; ●mono-adduct; ✶di-adduct*

The proportions of the different species in solution are drawn in Figure S4 (Figure 2 in the main text) as a function of the ratio $B_{10}H_9CO$/$SiW_{10}$-APTES, the ratio DIPEA/$B_{10}H_9CO$ being constant at 2. It evidences that it is possible to form quantitatively the di-adduct compound for an excess of $B_{10}H_9CO$ is used and reveals that the monoadduct is the major species (~80%) when $SiW_{10}$-APTES/$B_{10}H_9CO$/DIPEA = 1/1/2. To our knowledge, only di-adducts are known in the literature with POM-APTES systems.[8-13] This study by $^{29}Si$ NMR evidences that it could be possible to isolate selectively mono and di-adducts of $B_{10}H_9CO$ with $SiW_{10}$-APTES by playing on the proportions of the three reactants $SiW_{10}$-APTES, $B_{10}H_9CO$ and DIPEA in $CH_3CN$.

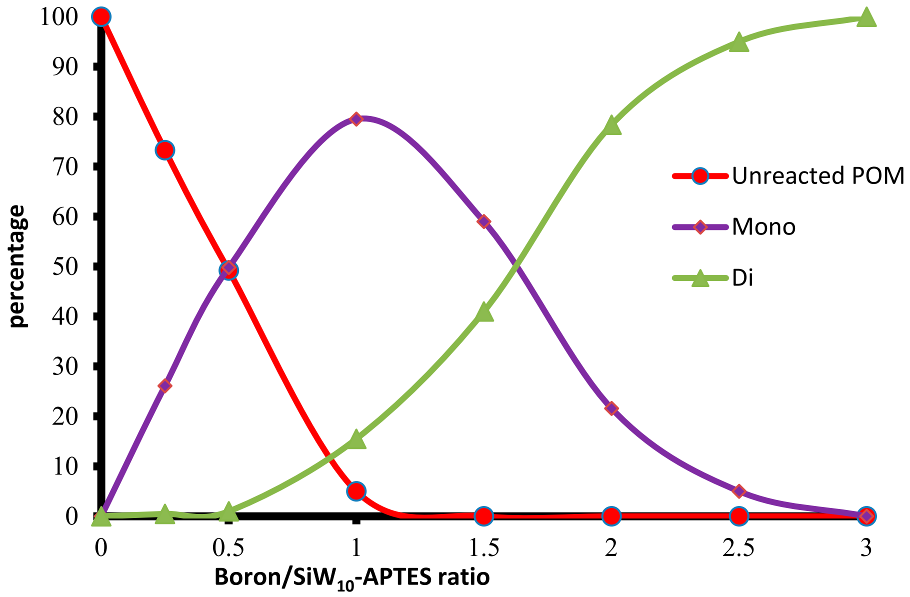


***Figure S4***: *Graph showing the evolution of the proportions of the products in the system $SiW_{10}$-APTES/$B_{10}H_9CO$/DIPEA as a function of $B_{10}H_9CO$/$SiW_{10}$-APTES ratio at fixed DIPEA/$B_{10}H_9CO$ ratio of 2.*

## $^1$H NMR Titrations

$^1$H nuclei are effective NMR probes for hybrids POMs, which allows getting spectra faster and performing 2D experiments. The tubes of the previous study were also analyzed by $^1$H NMR. As shown in Figure S5, the spectra are more complicated than the $^{29}$Si NMR spectra since they display the signals of the $TBA^+$ cations at 0.9, 1.4, 1.6, and 3.1 ppm, the signals of the $DIPEAH^+$ (1.3, 3.02, and 3.6 ppm), those of the solvents, and those of the $B_{10}H_9CO$ cluster core giving a very broad signal between +1.2 and -0.5 ppm[14] in addition to the protons of the three methylenic groups of the primary amine arms of APTES, which appear as small peaks on the spectra.

Despite of these difficulties, it is easy to identify the methylenic protons of the APTES chains denoted *a* and *c* on the picture of the Figure S5. The former correspond to the methylenic group in the vicinity of the Si atoms of APTES, while the latter are located in the alfa position of the terminal amine groups. In the starting precursor $SiW_{10}$-APTES broad signals located at 0.75, 1.91 and 3.30 ppm are assigned respectively to the protons *a*, *b*, and *c*, while a peak at 7.35 ppm is assigned to $NH_3^+$ functions in agreement with the literature. [8-11]

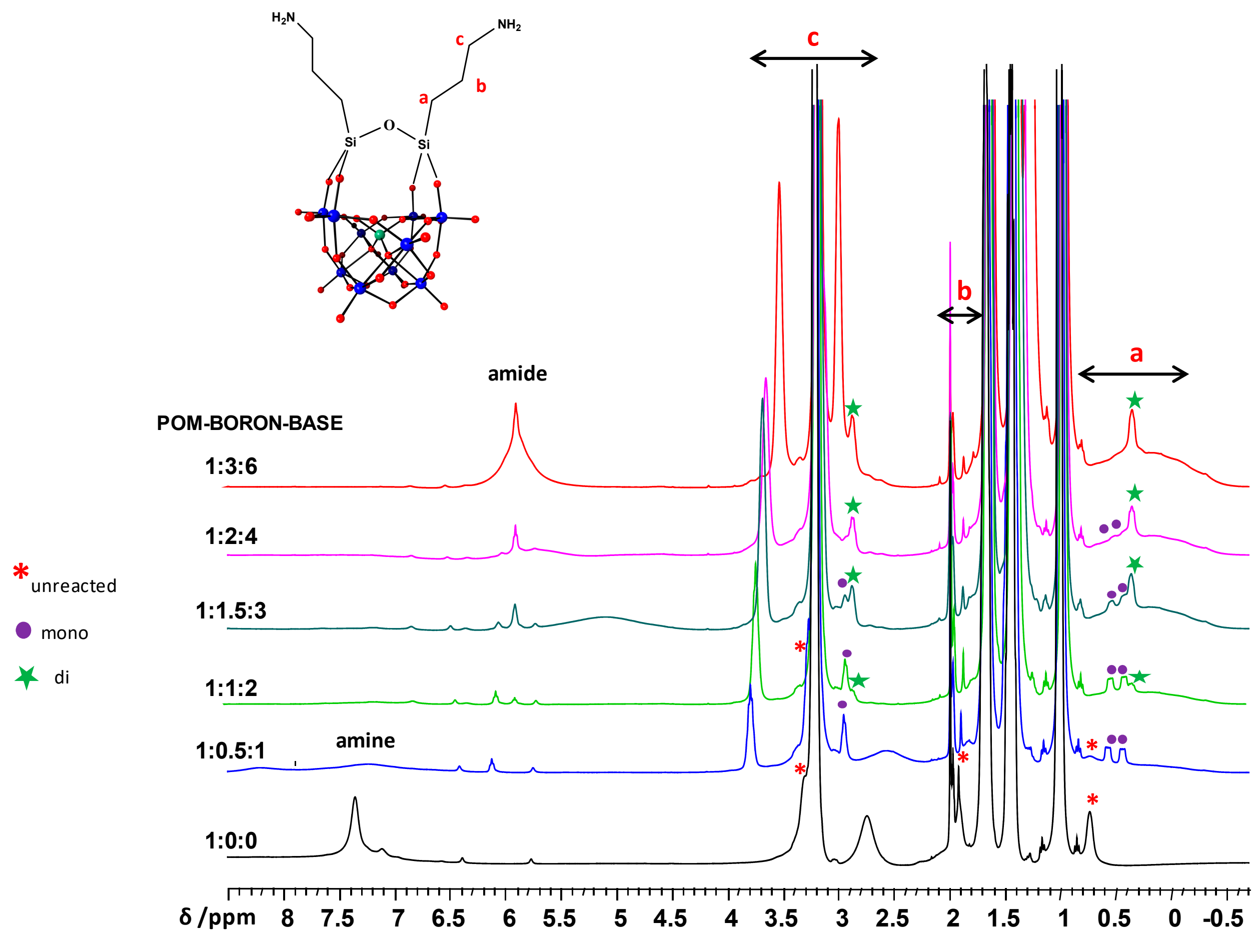


***Figure S5**: 400 MHz $^1H$ NMR spectra of the reaction medium with different $SiW_{10}$-APTES/$B_{10}H_9CO$/DIPEA ratios*

When the ratio $B_{10}H_9CO$/$SiW_{10}$-APTES increases, these two signals (*a* and *c*) disappear, while two new signals of the same intensities appear for the protons *a* at 0.45 and 0.58 ppm. These two signals agree with $^{29}Si$ NMR study and the formation of a mono-adduct compound in which the two alkyl chains of APTES are not equivalent. It is difficult to follow those corresponding to protons *c* and *b* due to overlapping with other signals. Increasing further the ratio $B_{10}H_9CO$/$SiW_{10}$-APTES leads to the decrease of these signals while a unique new signal appears at 2.86 ppm for protons *c* and at 0.35 ppm for protons *a* in agreement with the formation of a di-adduct derivative in which the two alkyl chains of the APTES are equivalent again. The peak of the free amine starts to disappear gradually as the ratio of Boron increases because of transformation into amide which appears at 6.15 ppm and becomes clearer at 5.9 ppm for the di adduct with total disappearance of the amine peak.

This study evidences that we can follow the formation of mono and di-adducts compounds formed by the reaction of $B_{10}H_9CO^-$ with $SiW_{10}$-APTES. Nevertheless, it remains difficult to estimate the fraction of the different species by $^1H$ NMR.

### 1.2.2 $P_2W_{17}$ derivatives.

Similar NMR studies were performed in solution with the Dawson derivative **$P_2W_{17}$-APTES**, which is also well known in the literature for the preparation of hybrid POMs.[15]

According to the previous study with $SiW_{10}$-APTES, we began by studying different mixtures with proportion $P_2W_{17}$-APTES/$B_{10}H_9CO$/DIPEA = 1/2/x, x being equal to 0, 2 or 4. Mixtures were prepared in $CD_3CN$ and $^{29}Si$, $^{31}P$ and $^{1}H$ NMR spectra were recorded. The $^{29}Si$ NMR spectra are given in Figure S5.

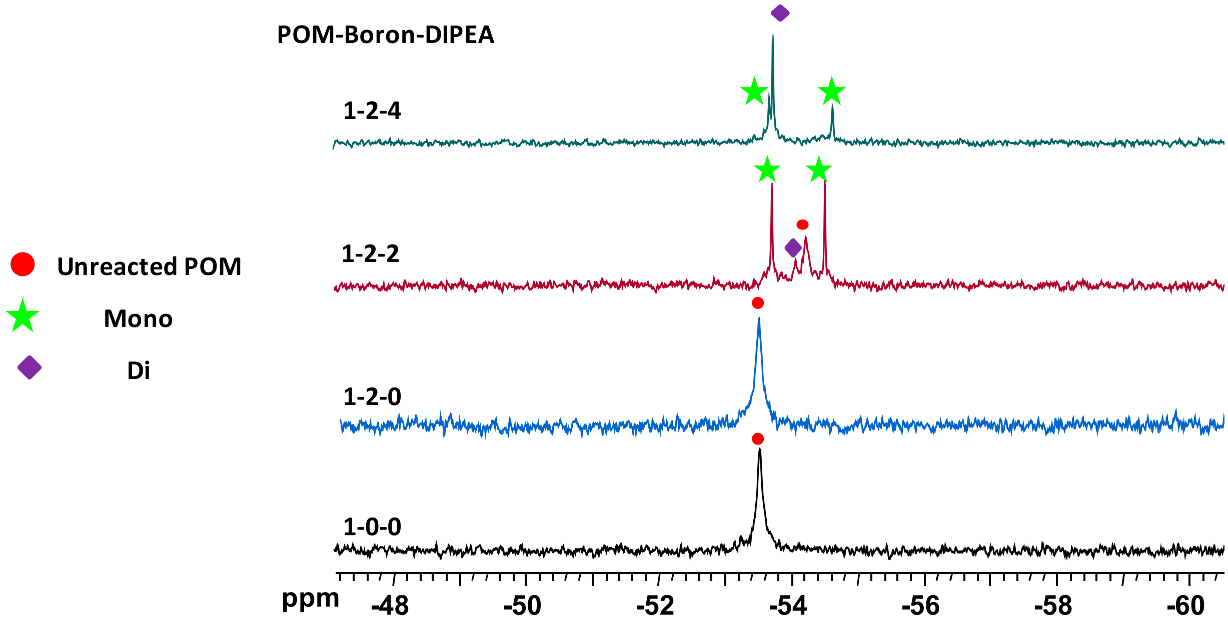


***Figure S6**: $^{29}Si$ NMR spectra from the reaction medium in case of $P_2W_{17}$-APTES with different DIPEA and boron cluster ratios in the system $P_2W_{17}$-APTES/$B_{10}H_9CO$/DIPEA.*

According to the previous studies, the Figure S6 evidences the important role that DIPEA plays in the reactivity with the $[B_{10}H_9CO]^-$ cluster but the conclusions are significantly different and the chemical shifts appear much more sensitive towards the addition of DIPEA compared to its $SiW_{10}$-APTES analogue, which make an accurate analysis of the $P_2W_{17}$-APTES/$B_{10}H_9CO$/DIPEA system difficult. Shortly, $^{29}Si$ NMR study evidences that without DIPEA, no reaction occurs, while increasing DIPEA equivalents keeping $P_2W_{17}$-APTES/$B_{10}H_9CO$ ratio constant, leads to a mixture of mono and di-adducts with a maximum proportion of around 60% for the mono adduct. In contrast, we were able to easily reach 80 % of the mono-adduct in the case of $SiW_{10}$-APTES system and until 100% with a fine tuning of the proportions of the reactants. $^{31}P$ NMR studies carried out on the same mixtures gave the same conclusions (see Figure S7).

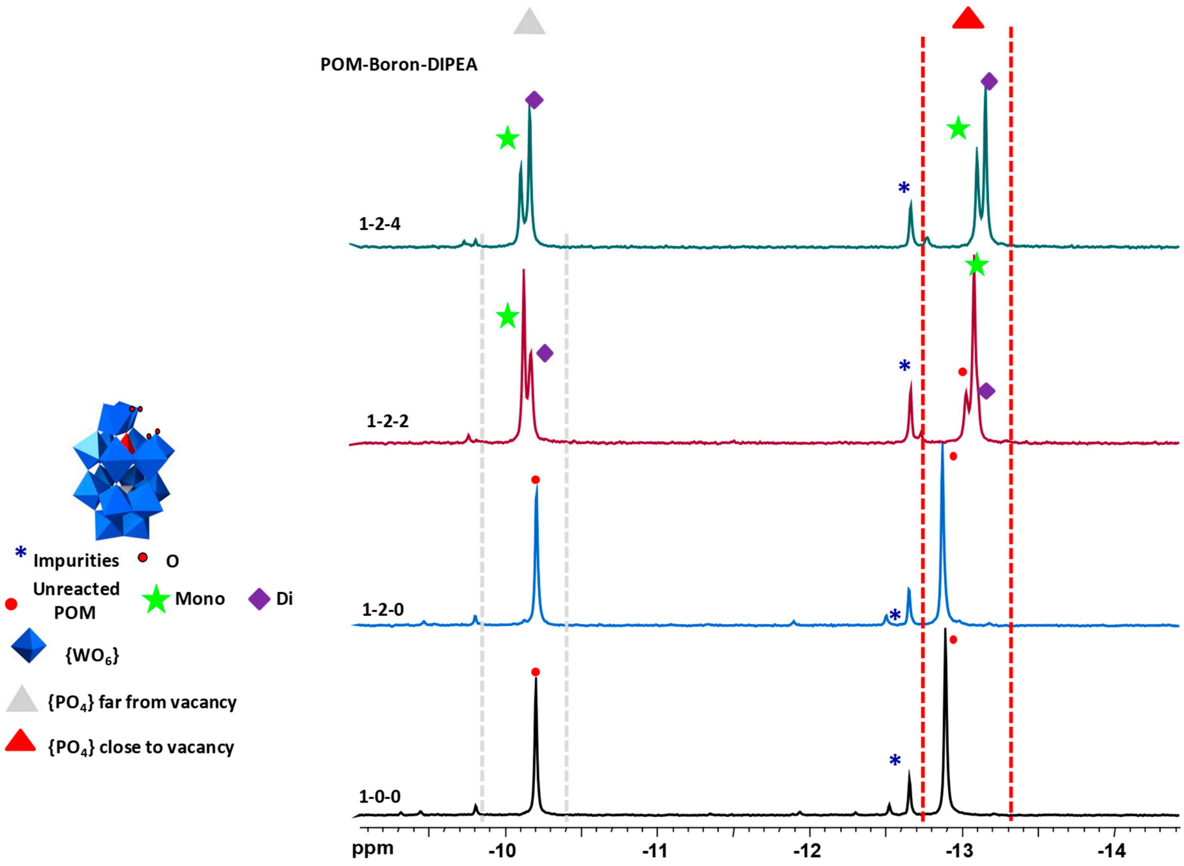


***Figure S7***: *$^{31}P$ NMR spectra for mixtures $P_2W_{17}$-APTES/$B_{10}H_9CO$/DIPEA at different ratios in $CD_3CN$.*

Given that, we decided to directly investigate the optimum ratios which were found with $SiW_{10}$-APTES. The corresponding reaction mixtures were prepared in $CD_3CN$ in closed flasks at room temperature and analyzed by $^{29}Si$, $^{31}P$ and $^{1}H$ NMR. The $P_2W_{17}$-APTES/$B_{10}H_9CO$/DIPEA mixtures in proportions 1/1/1.5, 1/1/2, and 1/3/6 were thus studied. Their $^{29}Si$ NMR spectra are given in Figure S8, while the $^{31}P$ and the $^{1}H$ NMR spectra are depicted in Figures S9 and S10 respectively, all in comparison with the corresponding spectra of the precursor $P_2W_{17}$-APTES.

.

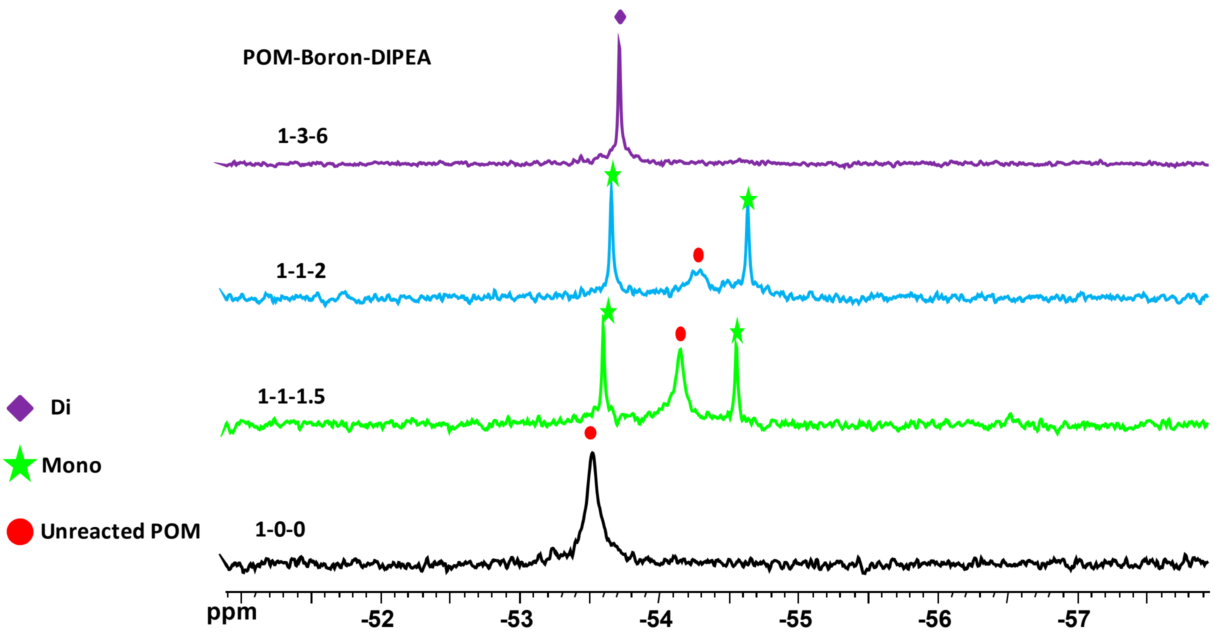


***Figure S8***: *$^{29}$Si NMR spectra for the reaction medium in the system $P_2W_{17}$-APTES/$B_{10}H_9CO$/DIPEA.*

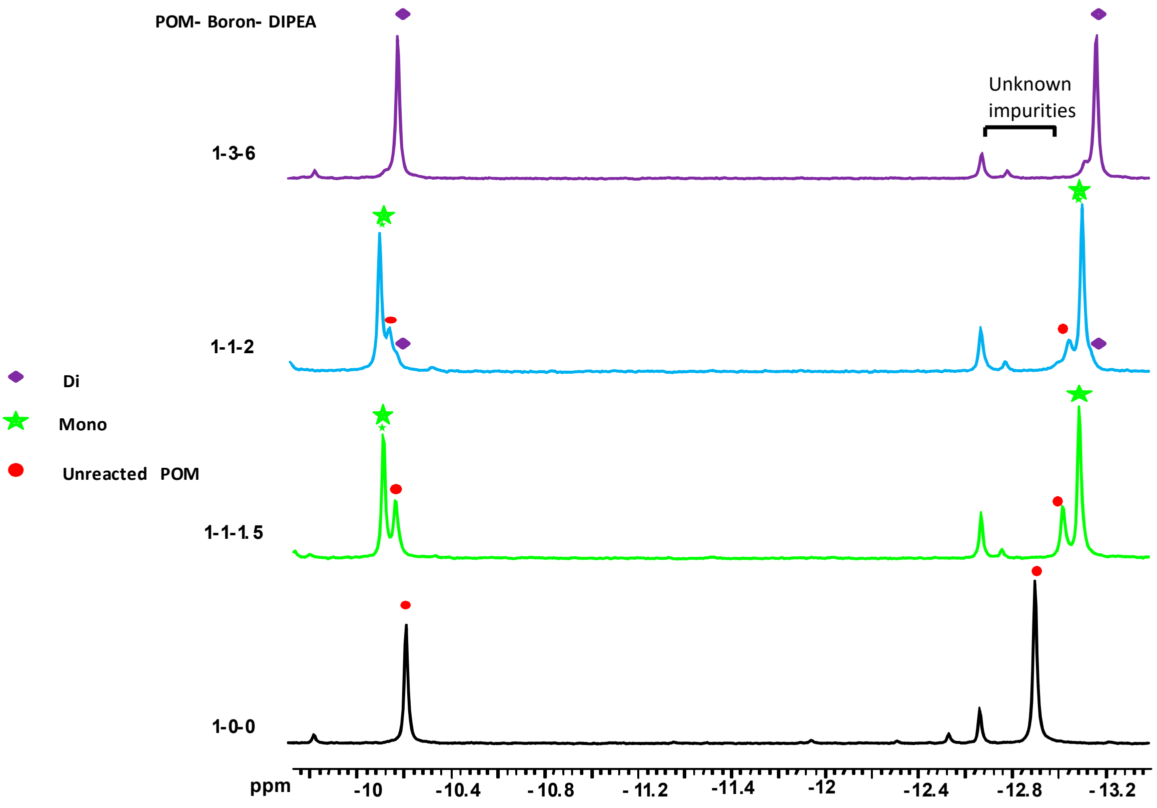


***Figure S9***: *$^{31}$P NMR spectra for the reaction medium in the system $P_2W_{17}$-APTES/$B_{10}H_9CO$/DIPEA.*

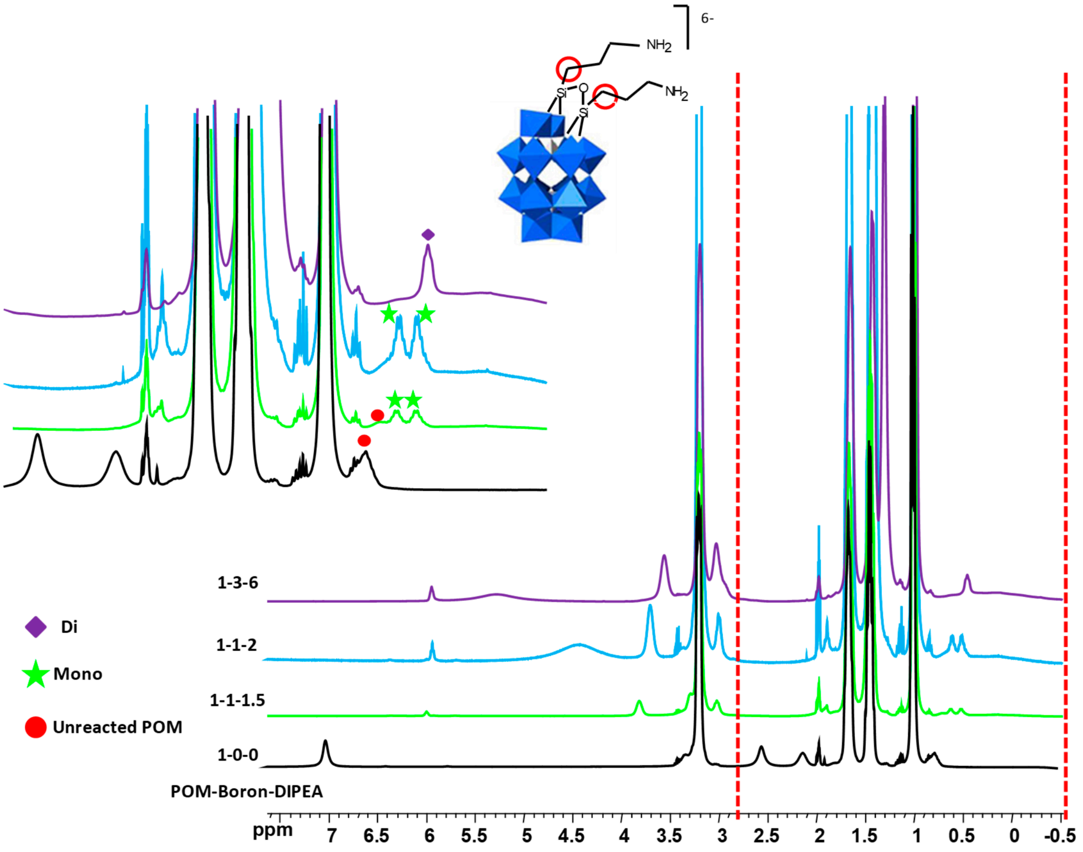


**Figure S10**: $^1$H NMR spectra for the reaction medium in the system $P_2W_{17}$-APTES/$B_{10}H_9CO$/DIPEA.

Both $^{29}$Si and $^{31}$P NMR spectra (Figures S8 and S9) showed that we have a mixture of mono adduct and unreacted POM for 1/1/1.5 and a mixture of mono-adduct, unreacted and small amount of di adduct for 1/1/2 proportions. This small amount was seen in the $^{31}$P NMR but overlapped in $^{29}$Si and $^1$H NMR appearing as broad peaks, while only one product, thus assigned to the pure di-adduct is seen for 1/3/6 mixture.

The $^1$H NMR spectra (Figure S10) agree with the proposed reaction scheme. As observed for the $SiW_{10}$-APTES series, the methylenic group in alfa position of the Si atoms of the APTES part appears characteristic of the species. For 1/1/1.5 and 1/1/2 ratios, two signals at 0.52 and 0.61 ppm are clearly observed for the mono-adduct in addition with a broad signal assigned to the starting POM precursor. In contrast the 1/3/6 mixture exhibit one signal at 0.45 ppm in agreement with the formation of a di-adduct product.

In summary, it is difficult to get a very high proportion of the mono-adduct without the starting compound or without the di-adduct compound. Consequently, we did not succeed to isolate this product. Conversely the di-adduct compound can be obtained quantitatively when the proportion $P_2W_{17}$-APTES/$B_{10}H_9CO$/DIPEA = 1/3/6 are used.

## 1.3 Syntheses of products

All reagents were purchased from commercial sources and used without further purification**.** All synthetic reactions were performed under Argon atmosphere using vacuum line and Schlenk-techniques. All solvents were dried and distilled unless stated otherwise, because of the sensitivity of the boron precursor used. $(NH_4)_2[B_{10}H_{10}]$ is provided by katchem company and $(TBA)_2[B_{10}H_{10}]$ was precipitated from an aqueous solution of $(NH_4)_2[B_{10}H_{10}]$ using saturated solution of TBABr, $TBA^+$ being tetrabutyl ammonium cations. $K_8(ß_2SiW_{11}O_{39}).14H_2O$, $K_8(\gamma\text{-}SiW_{10}O_{36}).12H_2O$, $K_6(\alpha\text{-}P_2W_{18}O_{62}).14H_2O$ and $K_{10}(\alpha\text{-}P_2W_{17}O_{61}).20H_2O$ were prepared according to the literature[16,17]. $TBA[B_{10}H_9CO]$ was prepared according to literature.[18]

**Synthesis of $(TBA)_3H[(\gamma\text{-}SiW_{10}O_{36})(Si(CH_2)_3NH_2)_2O].3H_2O$ , denoted $SiW_{10}$-APTES.**

The synthesis of **$SiW_{10}$-APTES** was adapted from Mayer's work.[19] $k_8(\gamma\text{-}SiW_{10}O_{36}).12H_2O$ (6 g, 2 mmol) was suspended with TBABr (1.94 g, 6 mmol) in a mixture of acetonitrile (60 mL) and water (16 mL). 3-aminopropyl triethoxy silane (0.94 mL, 4 mmol) and HCl (12 M, 1 mL) were successively added under vigorous stirring. The mixture was stirred for a further six hours. The white compound obtained after evaporation of the organic solution in a rotary evaporator was copiously washed with water, filtrated and dried by ether. Purification was done by re-dissolving the solid in 40 mL acetonitrile, centrifugation to remove insoluble residue, then evaporation of acetonitrile in vacuum to yield 5.47 g of the light yellow product (79.4 %). IR ($\nu/cm^{-1}$): 2962 (m), 2936 (m, sh), 2874 (m), 1625 (m, br), 1483 (m), 1380 (w), 1101 (m), 1042 (m, br), 961 (m), 901 (s), 887 (s), 819 (s), 780 (s), 736 (m, br). Elemental analysis for $(TBA)_3H[(\gamma\text{-}SiW_{10}O_{36})(Si(CH_2)_3NH_2)_2O].3H_2O$ ($C_{54}H_{131}N_5O_{40}Si_3W_{10}$, FW = 3413.4 $g.mol^{-1}$) Calc. (found): H 3.87 (3.84); C 19.00 (19.41); N 2.05 (2.04). TGA showed a weight loss of 1.45 % in the 20-250 °C temperature range corresponding to the hydration water (calculated 1.57 %) MALDI-TOF: *m/z* found 4092.3 (*m/z* calculated for $\{(TBA)_3H_2[(SiW_{10}O_{36})O(SiC_3H_6NH_2)_2](CH_3CN)_3(H_2O)_6(DCTB)_2\}^+$ 4092.3).

**Synthesis of $(TBA)_5H[\alpha2\text{-}P_2W_{17}O_{61}(NH_2CH_2CH_2CH_2Si)_2O]. 6H_2O$, denoted $P_2W_{17}$-APTES.**

A similar procedure was used for the synthesis of the precursor **$P_2W_{17}$-APTES**. "APTES", $NH_2CH_2CH_2CH_2Si(OCH_2CH_3)_3$ (410 µL, 1.76 mmol) was added dropwise to a solution of $H_2O/CH_3CN$ (80/40 mL v/v) at room temperature. Then solid $K_{10}\alpha\text{–}P_2W_{17}O_{61}.20H_2O$ (4 g, 0.8 mmol) was added to the solution which had been acidified to pH = 1.8 with 1 M HCl. The clear solution was stirred overnight. Then $CH_3CN$ was evaporated by rotary evaporator and $NBu_4Br$ (3.3 g, 10.4 mmol) was added to the aqueous solution to precipitate the expected product which was then washed copiously with water and kept for dryness. Purification was done by re-dissolving the solid in 30 mL acetonitrile, centrifugation to remove insoluble residue, then evaporation of acetonitrile in vacuum to yield 4.4 g of the light-yellow product (95 %). Further purification was done (when necessary) by diffusion with tert-butyl methyl ether into a solution of product in $CH_3CN$. IR ($\nu/cm^{-1}$): 2962 (m), 2936 (m, sh), 2874 (m), 1626 (m, br), 1484 (m), 1466 (m, sh), 1380 (w), 1087 (s), 1038 (w), 953 (s), 916 (s), 803 (s), 759 (sh, br), 719 (sh, br). Elemental analysis for $(TBA)_5H[\alpha\text{-}P_2W_{17}O_{61}(NH_2CH_2CH_2CH_2Si)_2O]. 6H_2O$

($C_{86}H_{209}N_7O_{68}P_2Si_2W_{17}$, FW = 5673.18 g.mol$^{-1}$) Calc. (found): H 3.71 (3.56); C 18.21 (17.96); N 1.73 (1.58). TGA showed a weight loss of 1.82 % in the 20-250 °C temperature range corresponding to the hydration water (calculated 1.9 %). $^{1}$H NMR (δ ppm, 400.13 MHz, $CD_3CN$): 0.79 (t, -$SiCH_2CH_2CH_2NH_2$), 2.13 (m, -$SiCH_2CH_2CH_2NH_2$), 3.56 (t, -$SiCH_2CH_2CH_2NH_2$), 7.02 (s, -$SiCH_2CH_2CH_2NH_2$), 3.22 (t, TBA), 1.66 (m,TBA), 1.45 (m, TBA), 1.00 (t, TBA). $^{13}$C{$^{1}$H} NMR (δ ppm, 100.62 MHz, $CD_3CN$): 10.8 (-$SiCH_2CH_2CH_2NH_2$), 22.4 (-$SiCH_2CH_2CH_2NH_2$), 43.5 (-$SiCH_2CH_2CH_2NH_2$), 59.4 (TBA), 24.6 (TBA), 20.6 (TBA), 14.2 (TBA). $^{29}$Si{$^{1}$H} NMR (δ ppm, 79.49 MHz, $CD_3CN$): -53.7 (1 Si of APTES). $^{183}$W NMR (δ ppm, 20.83 MHz, $CD_3CN$): -112.1 (2 W), -115.3 (2 W), -157.4 (2 W), -157.9 (1 W), -161.4 (2 W), -167.1 (2 W), -168 (2 W), -181.3 (2 W), -325 (2W). $^{31}$P NMR (δ ppm, 161.97 MHz, $CD_3CN$): -10.2 (1 P), -12.9 (1 P). ESI-MS (Negative mode, $CH_3CN$): *m/z* found (calculated), 1532.07 (*m/z* calculated for [[α2-$P_2W_{17}O_{61}(NH_2CH_2CH_2CH_2Si)_2O$]+TBA+2H]$^{3-}$ = 1532.07), 1612.54 (*m/z* calculated for [[α2-$P_2W_{17}O_{61}(NH_2CH_2CH_2CH_2Si)_2O$]+2TBA+H]$^{3-}$ = 1612.56), 2419.88 (*m/z* calculated for [[α2-$P_2W_{17}O_{61}(NH_2CH_2CH_2CH_2Si)_2O$]+2TBA+2H]$^{2-}$ = 2419.34). MALDI-TOF: *m/z* found = 6056.7 (*m/z* calculated for {(TBA)$_6$H[($P_2W_{17}O_{61}$)($NH_2C_3H_6Si)_2O$]($C_{17}H_{18}N_2)_1$}$^{+}$ = 6057.9).

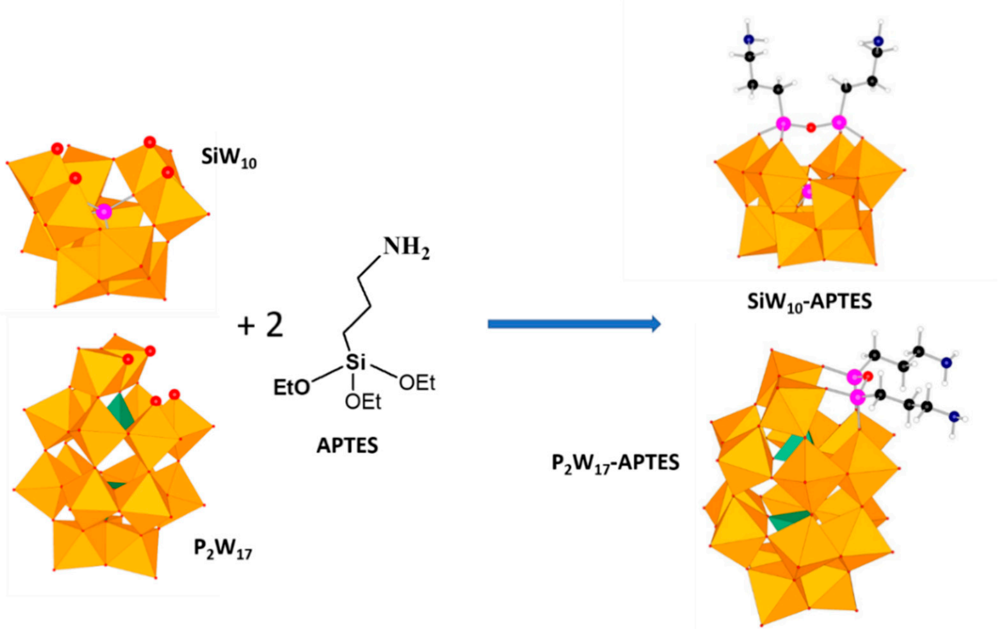


***Scheme S1.*** *Scheme of syntheses of POM-APTES precursors from lacunary POMs (left). The Nucleophilic O atoms of the POMs are highlighted by red dots. Two APTES molecules react with these oxygen atoms and establish a Si-O-Si bridge between them to provide the POM-APTES precursors used in this study. The reactions are performed in acetonitrile in presence of HCl. Legend: C in black, H in white, N in dark blue, Si in pink, O in red, B in blue, $WO_6$ octahedra in orange and $PO_4$ tetrahedra in green*

**Synthesis of (TBA)$_3$(DIPEAH)$_3$[($SiW_{10}O_{36}$)($B_{10}H_9CONHC_3H_6Si$)($NH_2C_3H_6Si$)O].3$H_2O$, denoted $SiW_{10}$-mono$B_{10}$.**

$SiW_{10}$-APTES (1 g, 0.29 mmol) was dissolved in 2 mL of dry acetonitrile, then TBA[$B_{10}H_9CO$] (113 mg, 0.29 mmol) was added with 1 mL of dry acetonitrile and stirred till total solubility.

DIPEA (76 µL, 0.44 mmol) was added drop by drop with vigorous stirring. The solution was kept at room temperature for 3 hours under Nitrogen. After the reaction time, the solution was kept under the hood for evaporation of acetonitrile phase. An oily residue was obtained and triturated by diethyl ether until a dry light yellow solid was obtained, filtrated, washed with diethylether for removing the excess of DIPEA and dried under vacuum for few hours to yield 1.07 g of $(C_{16}H_{36}N)_3(C_8H_{19}N)_3[(SiW_{10}O_{36})$ $(B_{10}H_9CONHC_3H_6Si)(NH_2C_3H_6Si)O](H_2O)_3$ (92.8 %). IR/cm$^{-1}$: 2961 (m), 2933 (m, sh), 2873 (m), 2469 (m), 1664 (sh), 1626 (m, br), 1482 (m), 1380 (w), 1099 (m), 959 (m), 899 (s), 884 (s), 819 (s), 733 (m, br). Elemental analysis for $B_{10}C_{79}H_{195}N_8O_{41}Si_3W_{10}$ (FW = 3944.31 g.mol$^{-1}$) Calc. (found): H 4.98 (4.83); C 24.06 (24.18); N 2.84 (2.12); B 2.74 (2.39); Si 2.14 (1.87). TGA showed a weight loss of 1.6 % in the 20-250 °C temperature range corresponding to the hydration water (calculated 1.4 %). MALDI-TOF: *m/z* found 3842.7 (*m/z* calculated for $\{(TBA)_4H_3[(SiW_{10}O_{36})(Si_2ON_2H_3C_6H_{12})(B_{10}H_9CO)](CH_3CN)_1(H_2O)_3\}^+$ 3843.1).

**Synthesis of $(TBA)_{6.5}(DIPEAH)_{1.5}[(SiW_{10}O_{36})(B_{10}H_9CONHC_3H_6Si)_2O].2H_2O$, noted $SiW_{10}$-di$B_{10}$.** $TBA_3H[(NH_2(CH_2)_3Si)_2O(\gamma\text{-}SiW_{10}O_{36}).3H_2O$ (0.5 g, 0.146 mmol) was dissolved in 1 mL of dry acetonitrile, then $TBA[B_{10}H_9CO]$ (0.17 g, 0.438 mmol) was added with 1 mL of dry acetonitrile and stirred till total solubility. DIPEA (152.8 µL, 0.876 mmol) was then added drop by drop with vigorous stirring. The solution was kept at room temperature for 3 hours under nitrogen. After the reaction time, the solution was kept under the hood for evaporation of acetonitrile phase. An oily residue was obtained and triturated by diethyl ether until a dry yellow solid was obtained, and filtrated. The product was washed many times with ethanol to remove excess of $TBAB_{10}H_9CO$ then dried under vacuum for few hours to yield 0.49 g of $(C_{16}H_{36}N)_{6.5}(C_8H_{19}N)_{1.5}(B_{10}H_9CONHC_3H_6Si)_2O(SiW_{10}O_{36})(H_2O)_2$ (71 %). IR/cm$^{-1}$: 3505 (w), 3412 (w), 2965 (m), 2936 (m, sh), 2875 (m), 2476 (s), 1670 (m), 1626 (m, br), 1482 (m), 1380 (w), 1099 (m), 961 (m), 947 (m), 899 (s), 884 (s), 838 (s), 820 (s), 745 (m, br). Elemental analysis for $B_{20}C_{124}H_{298.5}N_{10}O_{41}Si_3W_{10}$ (FW = 4725.25 g.mol$^{-1}$) Calc. (found): H 6.37 (6.66); C 31.52 (31.54); N 2.96 (2.78); B 4.58 (4.84); Si 1.78 (1.53). TGA showed a weight loss of 1 % in the 20-150 °C temperature range corresponding to the hydration water (calculated 0.76 %). MALDI-TOF: *m/z* found 4085.4 (*m/z* calculated for $\{(C_{16}H_{36}N)_4H_5[(SiW_{10}O_{36})(SiC_6H_{12}NHCOB_{10}H_9)_2O](CH_3CN)_2(H_2O)_6\}^+$ 4084.4).

**Synthesis of $(TBA)_6(DIPEAH)_4[(P_2W_{17}O_{61})(B_{10}H_9CONHC_3H_6Si)_2O].3H_2O_3$, noted $P_2W_{17}$-di$B_{10}$.** **$P_2W_{17}$-APTES** (0.5 g, 0.088 mmol) was dissolved in 1 mL of dry acetonitrile, then $TBA[B_{10}H_9CO]$ (102 mg, 0.264 mmol) was added with 1 mL of dry acetonitrile and stirred till total solubility. DIPEA (91.9 µL, 0.528 mmol) was added drop by drop with vigorous stirring. The solution was kept at room temperature for 3 hours under nitrogen. After the reaction time, the solution was kept under the hood for evaporation of acetonitrile phase. An oily residue was obtained and triturated by Diethyl ether until a dry yellow solid was obtained, and filtrated. The product was washed many times with ethanol to remove excess of $TBAB_{10}H_9CO$. Unknown impurities were removed by dissolving the product in acetonitrile and precipitating it by diffusion with tert-butyl methyl ether then the final pure product was filtered and dried under vacuum for

few hours to yield 0.54 g of $(C_{16}H_{36}N)_6(C_8H_{19}N)_4[(P_2W_{17}O_{61})(B_{10}H_9CONHC_3H_6Si)_2O].3H_2O$ (92 %). IR (ν/cm$^{-1}$): 3495 (w), 3421 (w), 2962 (m), 2936 (m, sh), 2874 (m), 2470 (s). 1628 (m), 1480 (m), 1457 (m, sh), 1381 (w), 1087 (s), 1036 (w), 951 (s), 914 (s), 797 (s), 754 (sh, br), 701 (sh, br). Elemental analysis for$B_{20}C_{136}H_{330}N_{12}O_{67}P_2Si_2W_{17}$ (FW = 6665.95 g.mol$^{-1}$) Calc. (found): H 4.99 (5.06); C 24.51 (24.33); N 2.52 (2.18); B 3.24 (3.61); Si 0.84 (0.87). TGA showed a weight loss of 0.95 % in the 20-150 °C temperature range corresponding to the hydration water (calculated 0.81 %). MALDI-TOF: *m/z* found 6084.10 (*m/z* calculated for $\{(C_{16}H_{36}N)_5H_6[(P_2W_{17}O_{61})(B_{10}H_9CONHC_3H_6Si)_2O](CH_3CN)_2(H_2O)_6\}^+$ 6048.49).

# Part 2. Characterization of adducts.

## 2.1 FT-IR spectra

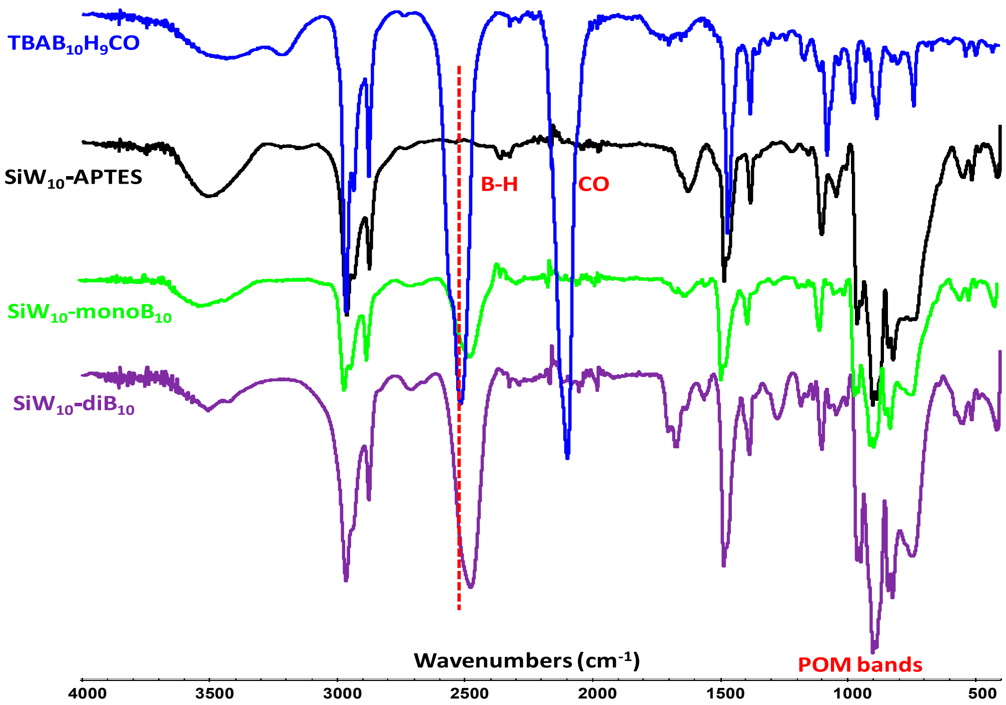


***Figure S11***: *IR spectra comparing TBA[$B_{10}H_9CO$], $SiW_{10}$-APTES, $SiW_{10}$-mono$B_{10}$, and $SiW_{10}$-di$B_{10}$. Dashed line represents a guide for the eyes to highlight the shift of the B-H bands*

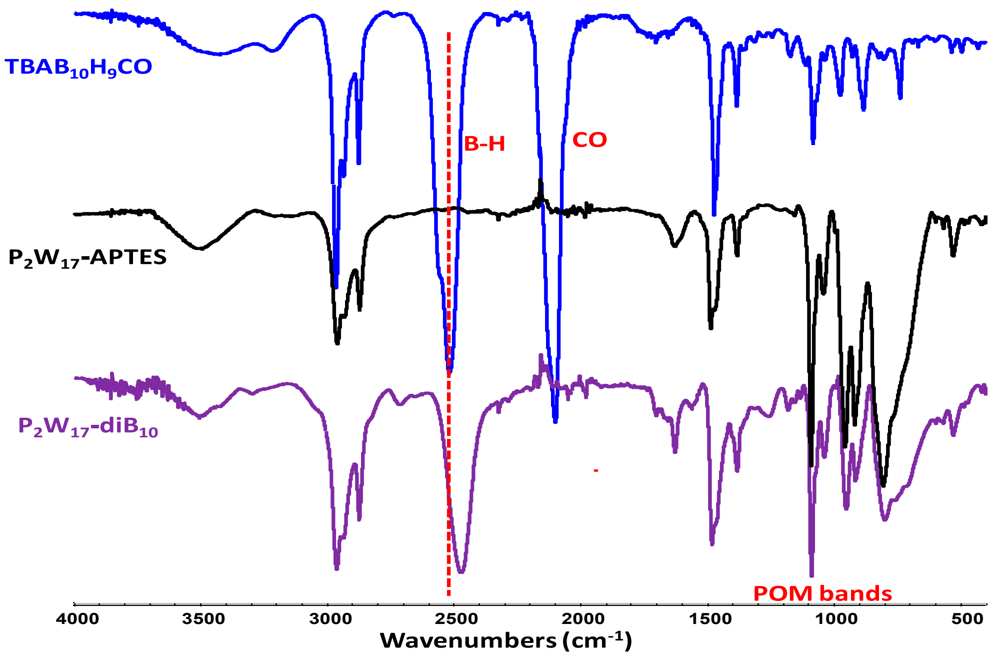


***Figure S12.*** *IR spectra comparing TBA[$B_{10}H_9CO$], $P_2W_{17}$-APTES, and $P_2W_{17}$-di$B_{10}$, dashed line represents a guide for the eyes to highlight the shift of the B-H bands*

## 2.2 MALDI-TOF data and spectra

***Table S1:*** **Summarized MALDI-TOF data**

| Compounds | Exp. *m/z* | Assignments | Calc. *m/z* |
|---|---|---|---|
| **$SiW_{10}$-APTES** | 4087.3 | $\{(TBA)_3H_2[(SiW_{10}O_{36})O(SiC_3H_6NH_2)_2](CH_3CN)_2(H_2O)_8(DCTB)_2\}^+$ | 4087.3 |
| | 4328.3 | $\{(TBA)_4H[(SiW_{10}O_{36})O(SiC_3H_6NH_2)_2](CH_3CN)_2(H_2O)_8(DCTB)_2\}^+$ | 4328.7 |
| **$SiW_{10}$-monoB$_{10}$** | 3843.2 | $\{(TBA)_4H_3[(SiW_{10}O_{36})O(SiC_3H_6NH_2)(SiC_3H_6NHCOB_{10}H_9)](CH_3CN)(H_2O)_3\}^+$ | 3843.1 |
| **$SiW_{10}$-diB$_{10}$** | 4084.8 | $\{(TBA)_4H_3[(SiW_{10}O_{36})O(SiC_3H_6NH_2)(SiC_3H_6NHCOB_{10}H_9)](CH_3CN)(H_2O)_3\}^+$ | 4084.4 |
| **$P_2W_{17}$-APTES** | 6058.7 | $\{(TBA)_6H[(P_2W_{17}O_{61})O(SiC_3H_6NH_2)_2](DCTB)\}^+$ | 6057.9 |
| | 6300.0 | $\{(TBA)_7[(P_2W_{17}O_{61})O(SiC_3H_6NH_2)_2](DCTB)\}^+$ | 6299.4 |
| **$P_2W_{17}$-diB$_{10}$** | 6048.1 | $\{(TBA)_5H_6[(P_2W_{17}O_{61})O(SiC_3H_6NHCOB_{10}H_9)_2](CH_3CN)_2(H_2O)_6\}^+$ | 6048.5 |
| | 6289.6 | $\{(TBA)_6H_5[(P_2W_{17}O_{61})O(SiC_3H_6NHCOB_{10}H_9)_2](CH_3CN)_2(H_2O)_6\}^+$ | 6290.1 |

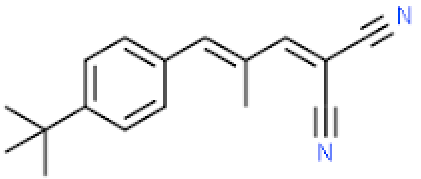


**= DCTB**

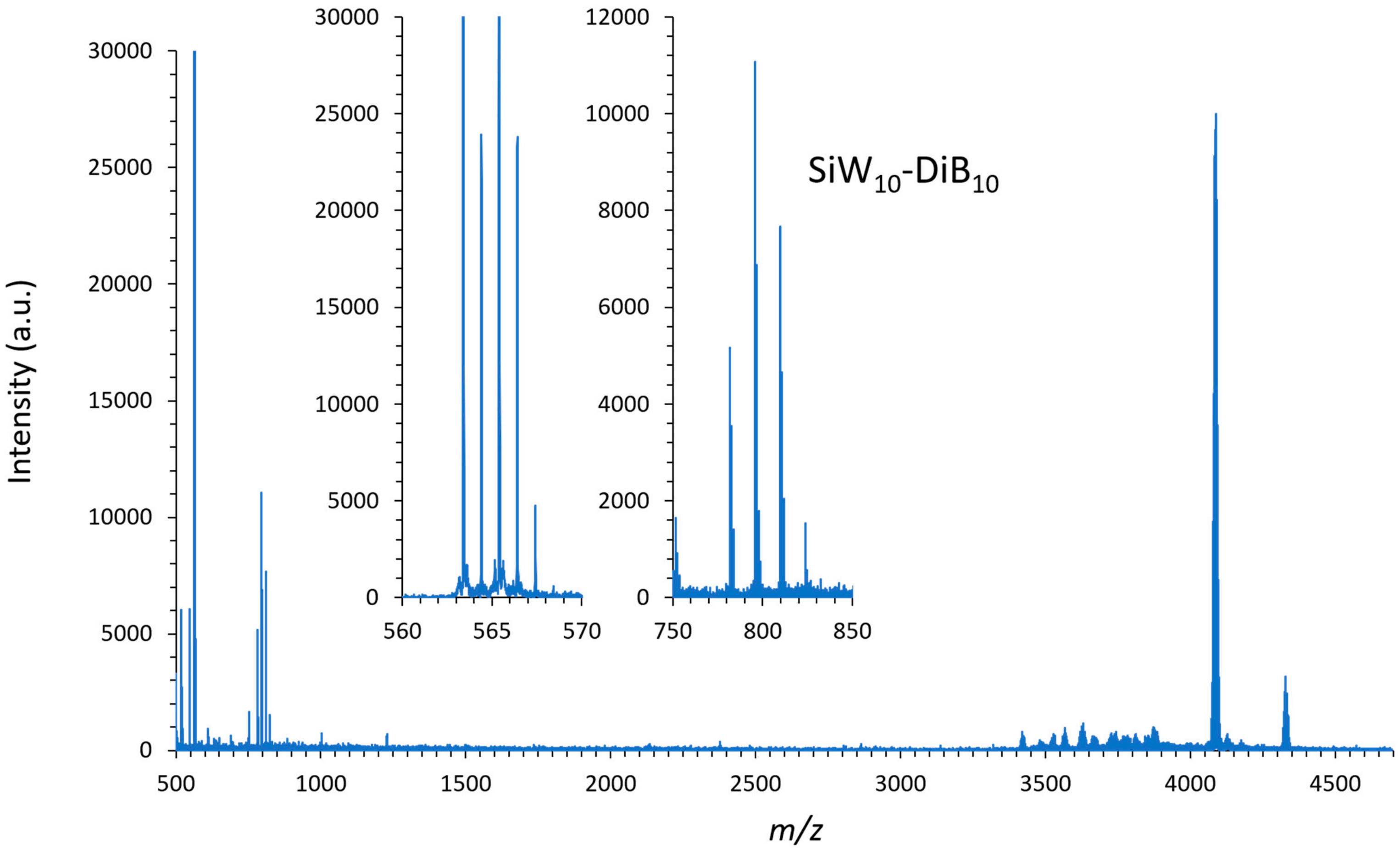


***Figure S13*.** Example of full MALDI-TOF spectrum recorded in positive mode in the 500-4700 m/z range for compound **$SiW_{10}$-diB$_{10}$**. Decomposition products between m/z 500 and m/z 850 can be seen for isotopic massifs corresponding to monocationic species, which do not contain W atoms. From the profile it could contain B atoms but the species were not identified. No species are detected between m/z 850 and m/z 3300. Considering that a full spectra in high resolution contains at least 400000 data, the other spectra were recorded in a smaller range, usually from m/z 2000.

## MALDI-TOF Spectra of $SiW_{10}$-$DiB_{10}$

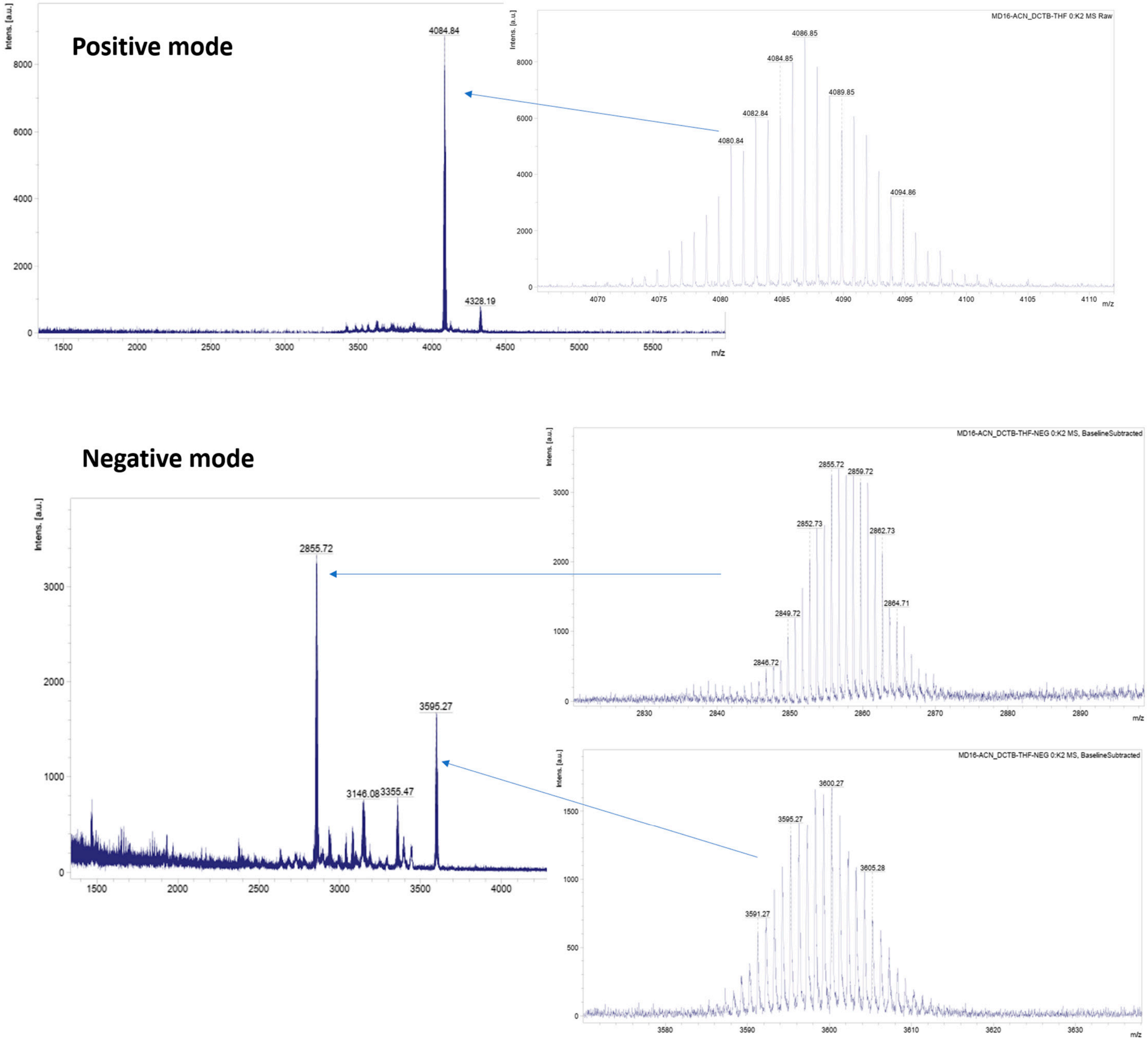


***Figure S14***. Comparison of MALDI-TOF spectra of compound **$SiW_{10}$-$diB_{10}$** recorded in negative and in positive modes. In the negative mode, more peaks are observed and the main peaks are assigned to degraded compounds.

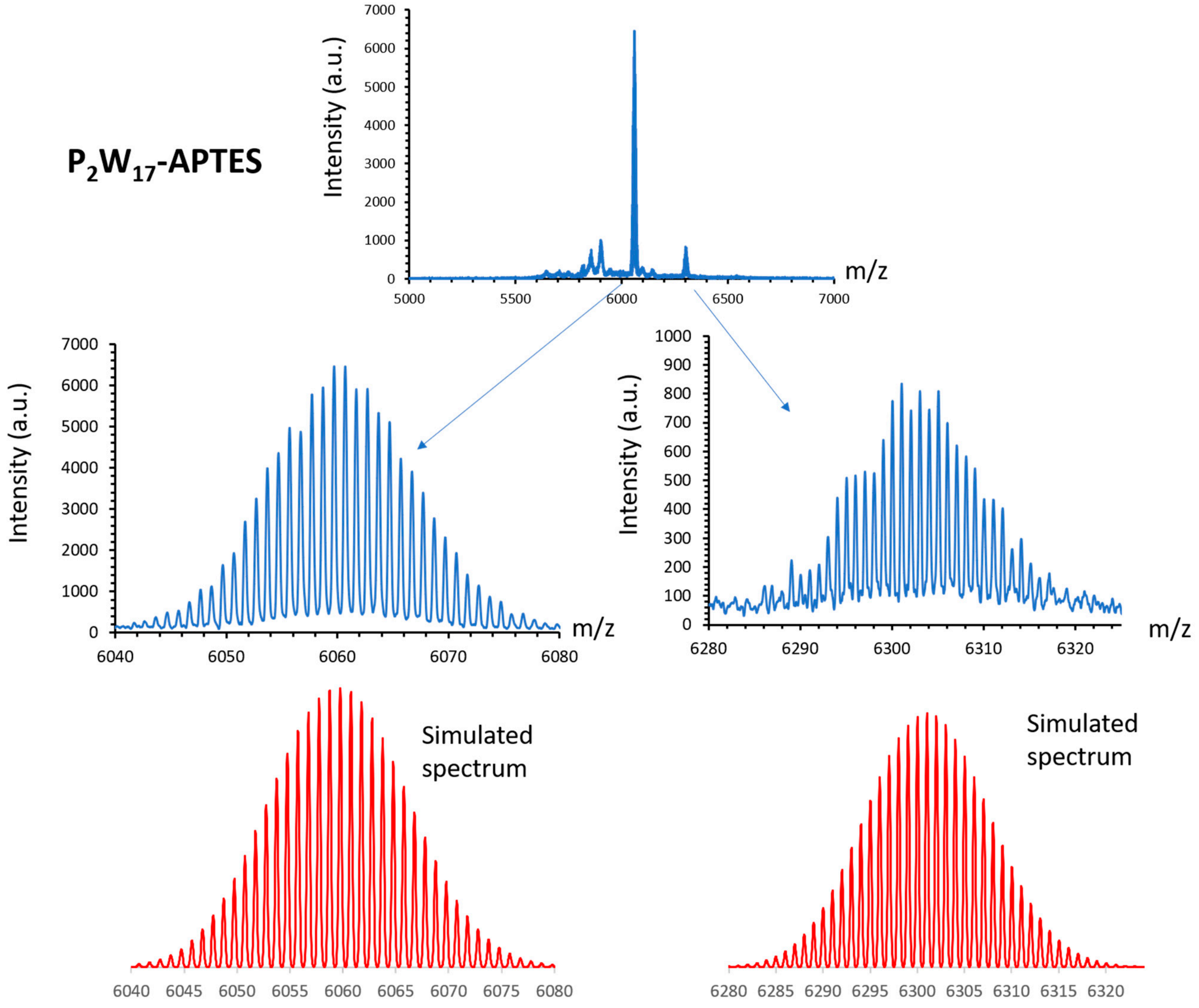


**Figure S15**. Experimental and simulated positive ion MALDI-TOF spectra of $P_2W_{17}$-APTES. Simulations (in red) have been performed with ISOPRO3 software for the formulas $(TBA)_6H[(P_2W_{17}O_{61})O(SiC_3H_6NH_2)_2](DCTB)\}^+$ (left) and $(TBA)_7[(P_2W_{17}O_{61})O(SiC_3H_6NH_2)_2](DCTB)\}^+$ (right).

## Experimental spectra of $P_2W_{17}$-APTES-Di$B_{10}$

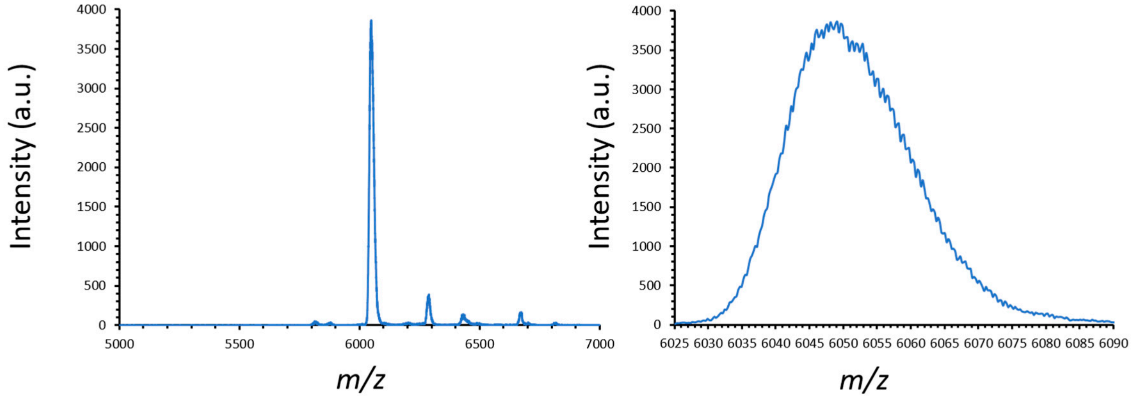


Simulated spectra for species $\{(TBA)_5H_6[(P_2W_{17}O_{61})O(SiC_3H_6NHB_{10}H_9CO)_2](CH_3CN)_x(H_2O)_y\}^+$ *with x ranging from 1 to 5 and y from 0 to 8.*

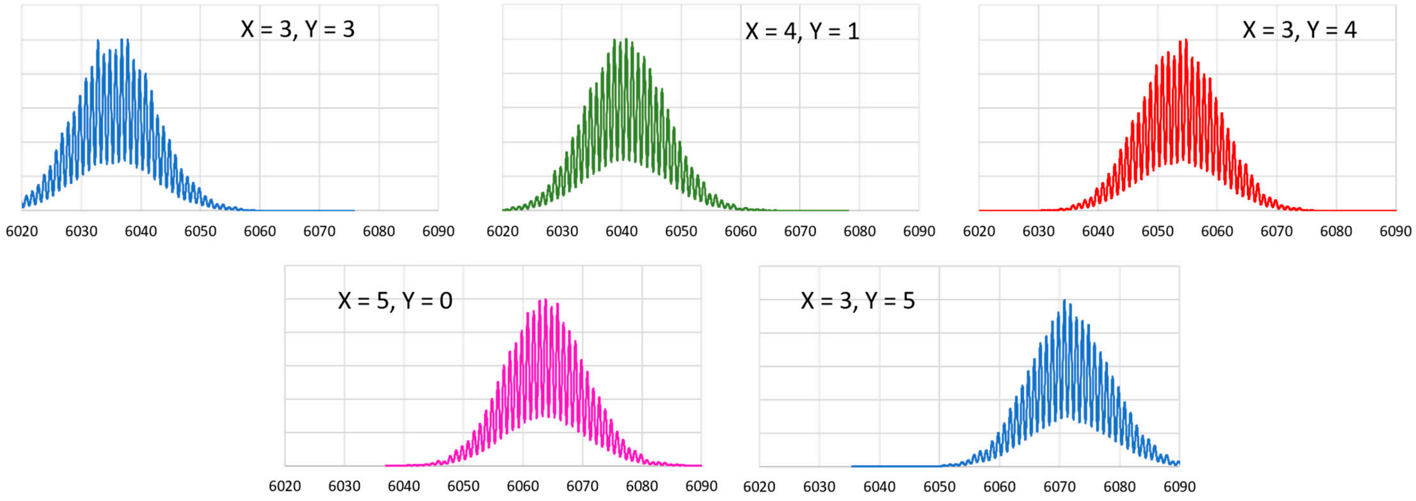


***Figure S16**. Linear positive ion MALDI-TOF spectrum of $P_2W_{17}$-di$B_{10}$ (up part) and simulated spectra of different species of general formula* $\{(TBA)_5H_6[(P_2W_{17}O_{61})O(SiC_3H_6NHCOB_{10}H_9)_2](CH_3CN)_x(H_2O)_y\}^+$ *with x ranging from 1 to 5 and y from 0 to 8*

## 2.3 NMR characterizations

### Table S2. NMR data

| Experiment | Compounds | Main Chemical shifts /ppm (signals of $TBA^+$ and $DIPEAH^+$ are not given) |
|---|---|---|
| $^{1}H\{^{11}B\}^{a}$ | $SiW_{10}$-APTES | 0.74 (*a*), 1.92 (*b*), 3.30 (*c*), 7.35 (*d*) |
| | $SiW_{10}$-mono$B_{10}$ | 0.43 (*a*), 0.56 (*a'*), 1.69 (*b*), 1.38 (*b'*), 2.94 (*c*), 2.93 (*c'*), 5.70 (*d'*, amine frozen), 6.12 (*d* amide), 6.33 (d', amine frozen), 7.24 (*d'*amine free) |
| | $SiW_{10}$-di$B_{10}$ | 0.38 (*a*), 1.97 (*b*), 2.89 (*c*), 5.94 (*d*) |
| | $P_2W_{17}$-APTES | 0.79 (*a*), 2.13 (*b*), 3.56 (*c*), 7.02 (*d*) |
| | $P_2W_{17}$-di$B_{10}$ | 0.45 (*a*), 1.97 (*b*), 2.95 (*c*), 5.94 (*d*) |
| $^{13}C\{^{1}H\}^{a}$ | $SiW_{10}$-APTES | 13.2 (*a*), 22.9 (*b*), 44.4 (*c*) |
| | $SiW_{10}$-mono$B_{10}$ | 13.9 (a), 14.1 (*a'*), 25.6 (*b*), 22.6 (*b'*), 42.3 (*c*), 44.1 (*c'*), 203.7 (CO) |
| | $SiW_{10}$-di$B_{10}$ | 13.8 (*a*), 25.6 (*b*), 42.5 (*c*), 203.0 (CO) |
| | $P_2W_{17}$-APTES | 10.8 (*a*), 22.4 (*b*), 43.5 (*c*) |
| | $P_2W_{17}$-di$B_{10}$ | 12.1 (*a*), 24.9 (*b*), c: 42.4 (*c*), 203.0 (CO) |
| $^{29}Si\{^{1}H\}$ | $SiW_{10}$-APTES | -62.5 (2Si), -88.2 (1Si) |
| | $SiW_{10}$-mono$B_{10}$ | -61.6 (1Si), -63.3 (1 Si), -88.4 (1Si) |
| | $SiW_{10}$-di$B_{10}$ | -62.3 (2Si), -88.6 (1Si) |
| | $P_2W_{17}$-APTES | -53.7 (1Si) |
| | $P_2W_{17}$-di$B_{10}$ | -53.7 (1Si) |
| $^{11}B\{^{1}H\}$ | $TBAB_{10}H_9CO$ | 6.2 (1B), 5.4 (1B), -18.3 (1B), -26.5 (2B), -28.9 (2B), -29.4 (2B), -44.4 (1B) |
| | $SiW_{10}$-mono$B_{10}$ | -0.0 (1 B), -0.8 (1B), -25.6 (1B), -28.0 (7B) |
| | $SiW_{10}$-di$B_{10}$ | -0.2 (d, 1B), -0.6 (d, 1B), -25.6 (s, 1B), -26 to -32 (br, 7B) |
| | $P_2W_{17}$-di$B_{10}$ | -0.2 (d, 1B), -0.6 (d, 1B), -25.6 (s, 1B), -26 to-32 (br, 7B) |
| $^{183}W$ | $SiW_{10}$-APTES | -108.6 (4W), -133.3 (2W), -141.9 (4W)<br>-137.2 (2 W), -143.1 (2 W), -143.7 (2 W) |
| | $SiW_{10}$-mono$B_{10}$ | -107.4 (2 W), -108.2 (2W), -137.2 (2W), -143.1 (2W), -143.7 (2W) |
| | $SiW_{10}$-di$B_{10}$ | -107.7 (4W), -137.1 (2W), -143.5 (4W) |
| | $P_2W_{17}$-APTES | -112.1 (2W), -115.3 (2W), -157.4 (2W), -157.9 (1W), -161.4 (2W), -167.1 (2W)<br>-168.0 (2W), -181.3 (2W), -325.0 (2W) |
| | $P_2W_{17}$-di$B_{10}$ | -117.1 (2W), -160.3 (2W), -164.2 (2W), -167.7 (1W), -168.0 (2W), -175.9 (2W), -183.2 (2W), -203.5 (2W), -324.5 (2W) |
| $^{31}P$ | $P_2W_{17}$-APTES | -10.2 (1 P), -12.9 (1 P) |
| | $P_2W_{17}$-di$B_{10}$ | -10.2 (1 P), -13.2 (1 P) |

[a] Assignments in $^{1}H$ and $^{13}C$ NMR: *a*, *b*, *c*, and *d refer to APTES protons, Si-$CH_2$-$CH_2$-$CH_2$-$NH_2$,* respectively. In case of mono adduct, *a'*, *b'*, *c'*, and *d' refer to corresponding APTES protons non-bonded to boron cage*. These assignments are based on $^{1}H$-$^{1}H$ COSY, $^{1}H\{^{13}C\}$ HMBC, and $^{1}H\{^{15}N\}$ HMBC.

## $^{11}$B NMR experiments

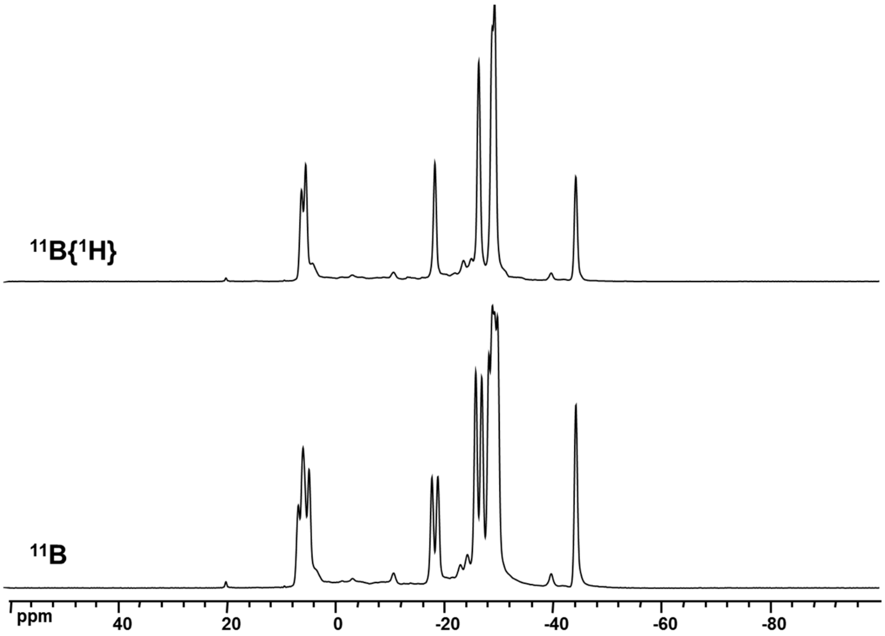


***Figure S17:*** *$^{11}$B and $^{11}$B {$^1$H} NMR spectra of TBA$B_{10}H_9$CO in $CD_3CN$.*

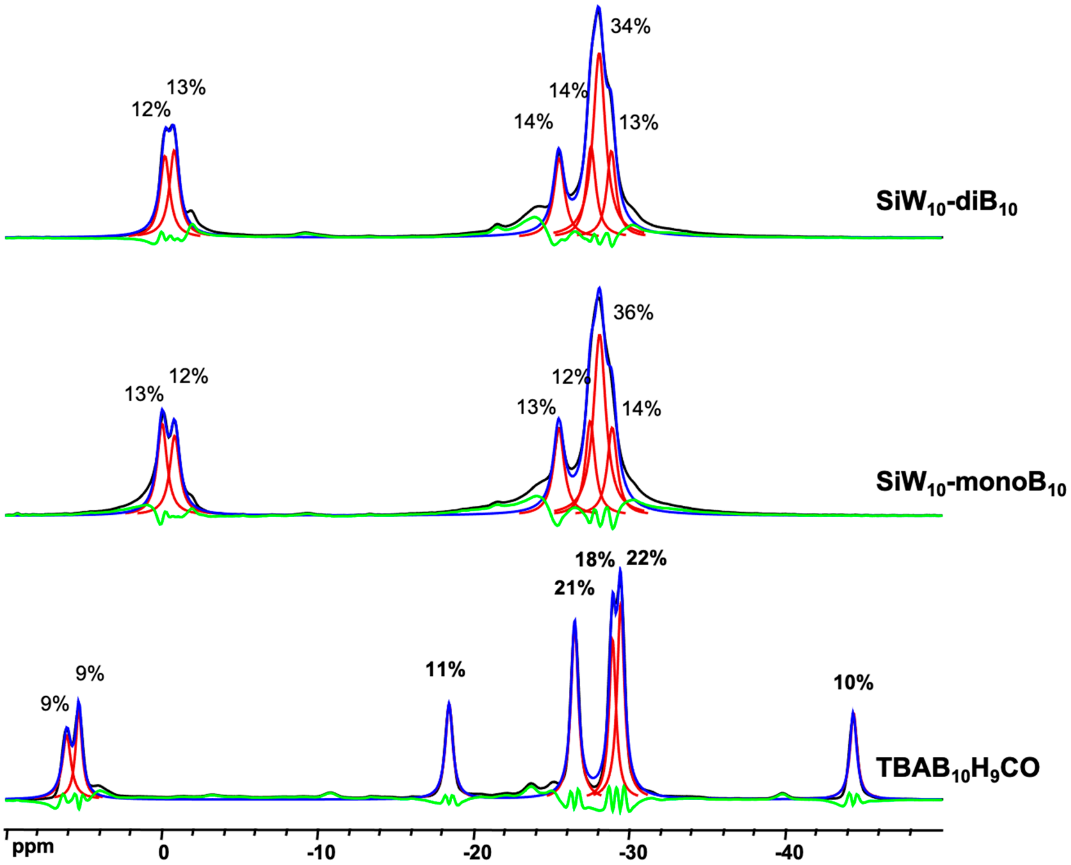


***Figure S18:*** *Spectral decomposition of* $^{11}$B{$^1$H} NMR of TBA[$B_{10}H_9$CO], $SiW_{10}$-mono$B_{10}$, and $SiW_{10}$-di$B_{10}$ showing the sites distribution of 1B:1B:1B:2B:2B:2B:1B in TBA[$B_{10}H_9$CO], and of 1B:1B:1B:(7B) in $SiW_{10}$-mono$B_{10}$, and $SiW_{10}$-di$B_{10}$. Black line: experimental, Blue line: calculated, green line: difference, red lines: components.

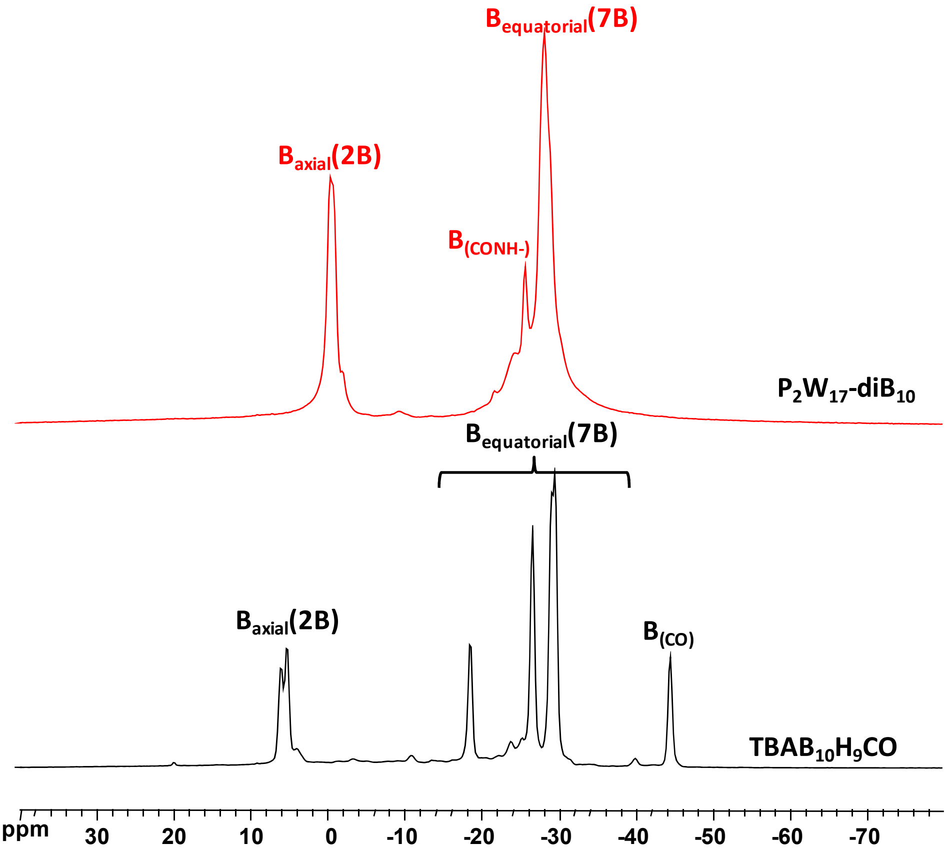


***Figure S19:*** *$^{11}B\{^1H\}$ NMR spectra of $P_2W_{17}$-diB$_{10}$ and TBA[$B_{10}H_9CO$] in $CD_3CN$.*

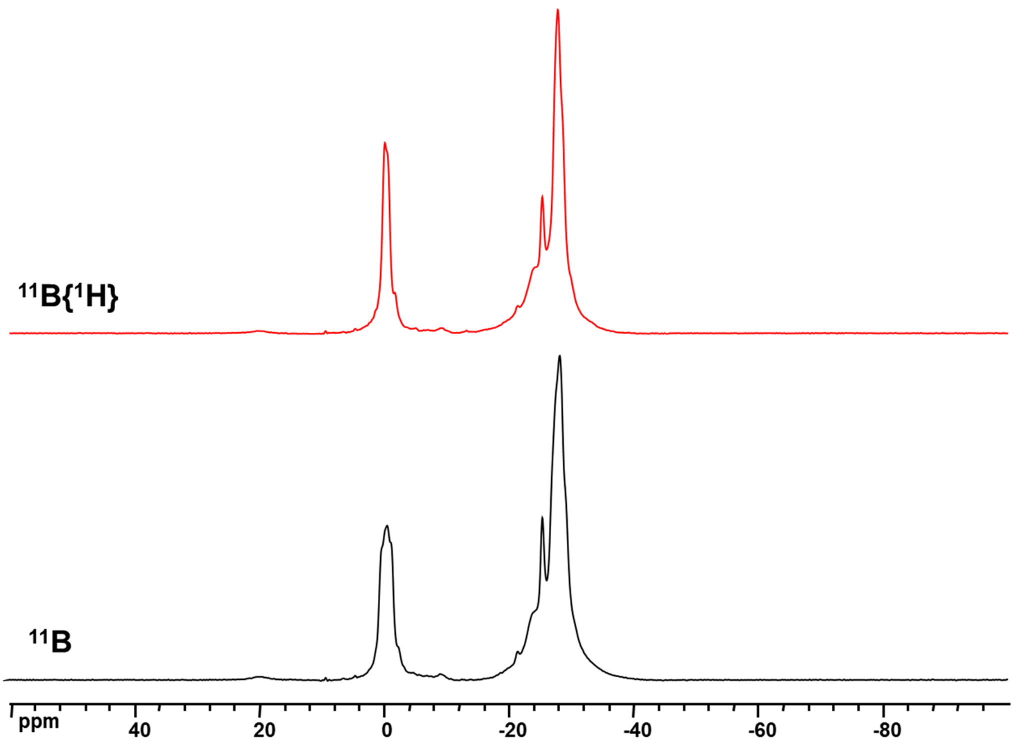


***Figure S20:*** *$^{11}B$ and $^{11}B\{^1H\}$ NMR spectra of $P_2W_{17}$-diB$_{10}$ in $CH_3CN$*

## $^{29}Si$ NMR experiments

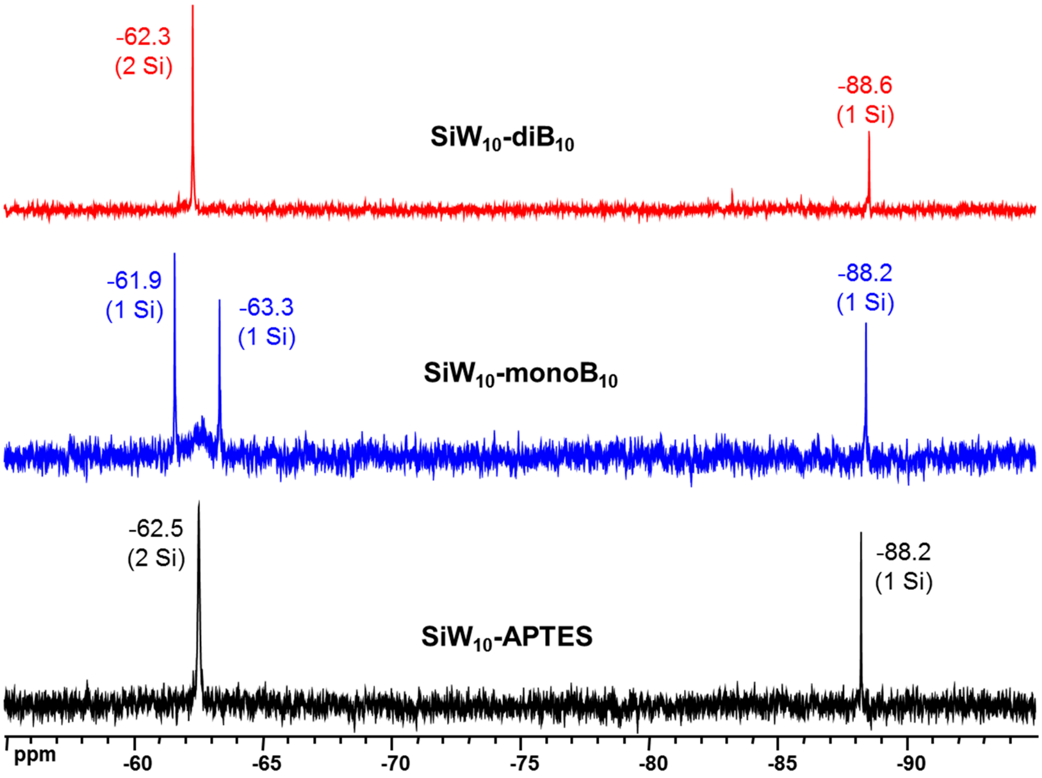


***Figure S21***. *$^{29}Si\{^1H\}$ NMR spectra of $SiW_{10}$-monoB$_{10}$, $SiW_{10}$-diB$_{10}$ and $SiW_{10}$-APTES in $CD_3CN$*

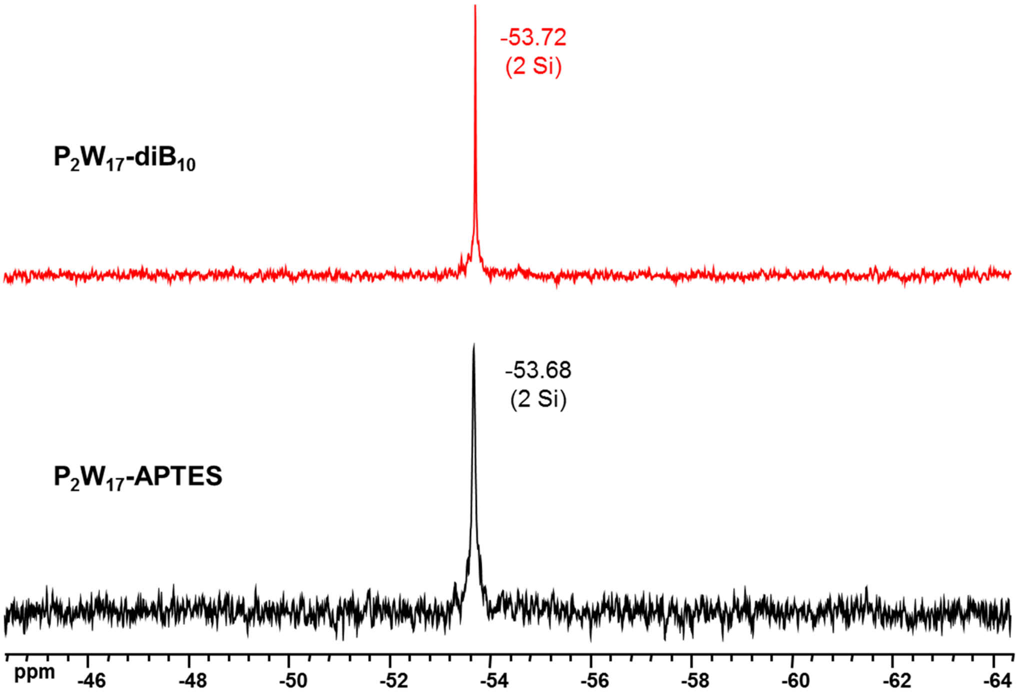


***Figure S22.*** *$^{29}Si\{^1H\}$ NMR spectra of $P_2W_{17}$-diB$_{10}$ and $P_2W_{17}$-APTES in $CD_3CN$.*

## $^{31}P$ NMR experiments

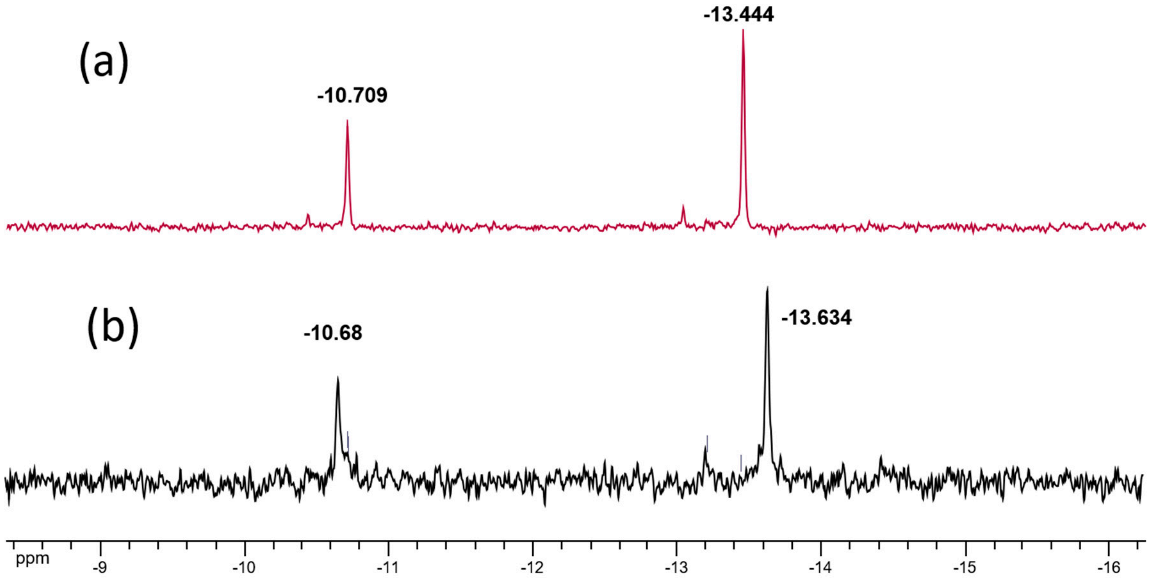


***Figure S23.*** *$^{31}P$ NMR spectra of $P_2W_{17}$-APTES (a) and $P_2W_{17}$-diB$_{10}$ (b) in $CD_3CN$. Unknown impurities were observed in $^{31}P$ NMR but not in the spectra of other NMR probes.*

## $^{1}H$ NMR experiments / 2D experiments ($^{1}H$ COSY, $^{1}H$ ROESY, $^{1}H$-$^{15}N$ HMBC)

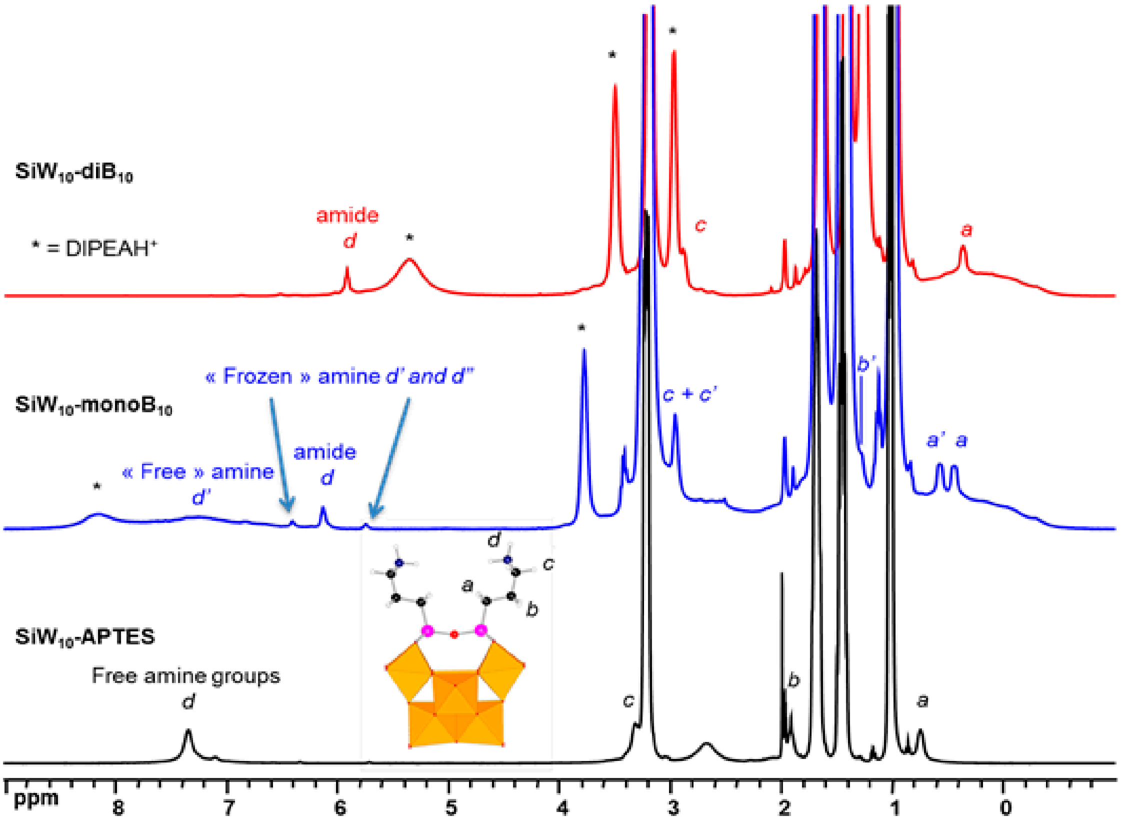


**Figure S24.** $^{1}H$ NMR spectra of $SiW_{10}$-mono$B_{10}$, $SiW_{10}$-di$B_{10}$ and $SiW_{10}$-APTES in $CD_3CN$

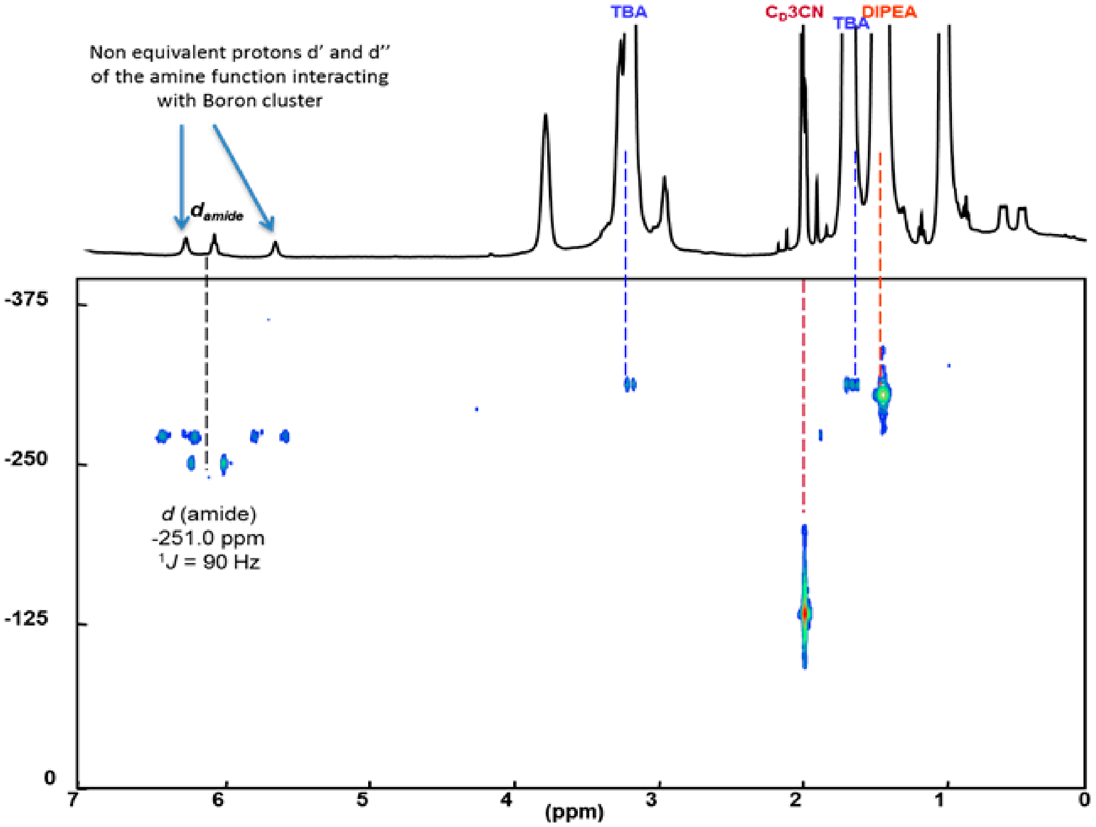


**Figure S25** *$^{1}H$-$^{15}N$ HMBC NMR spectrum of$SiW_{10}$-mono$B_{10}$ in $CD_3CN$*

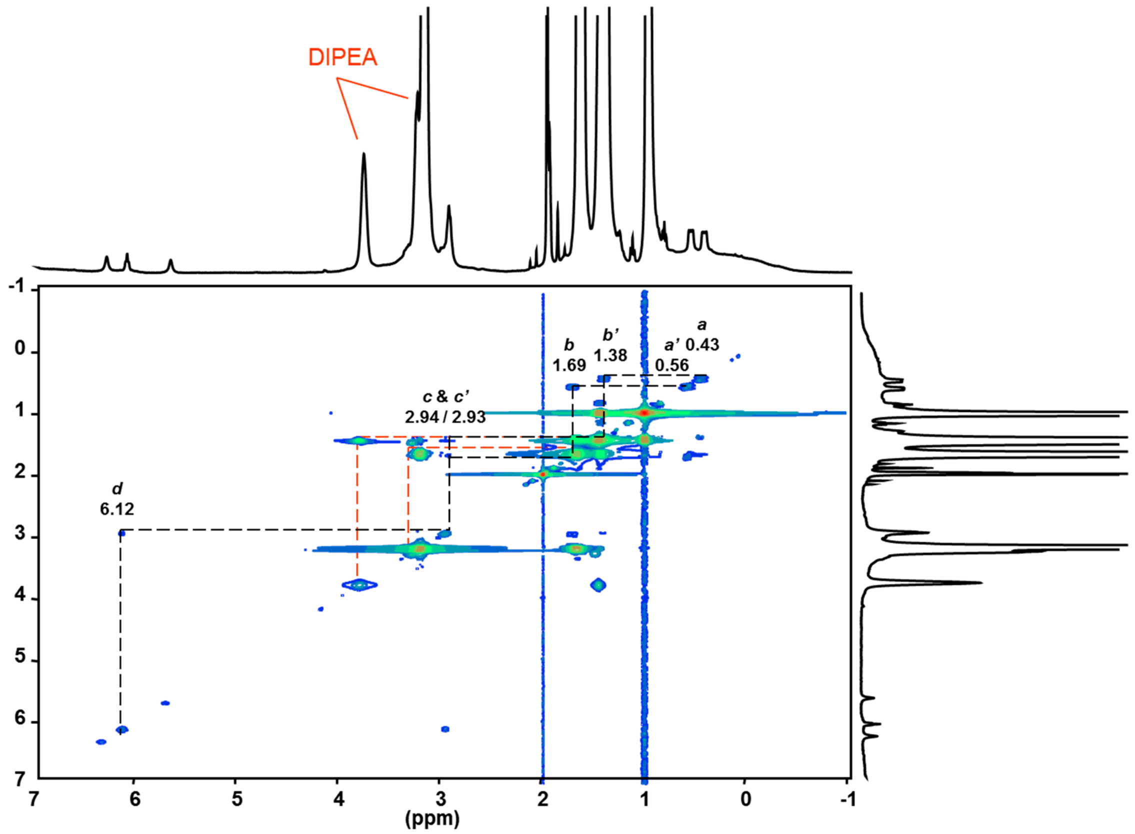


**Figure S26.** 2D 1H COSY NMR spectrum of $SiW_{10}$-mono$B_{10}$ in $CD_3CN$ allowing full assignment of all signals and identifying overlapped signals b, b', c, and c' of the APTES linker

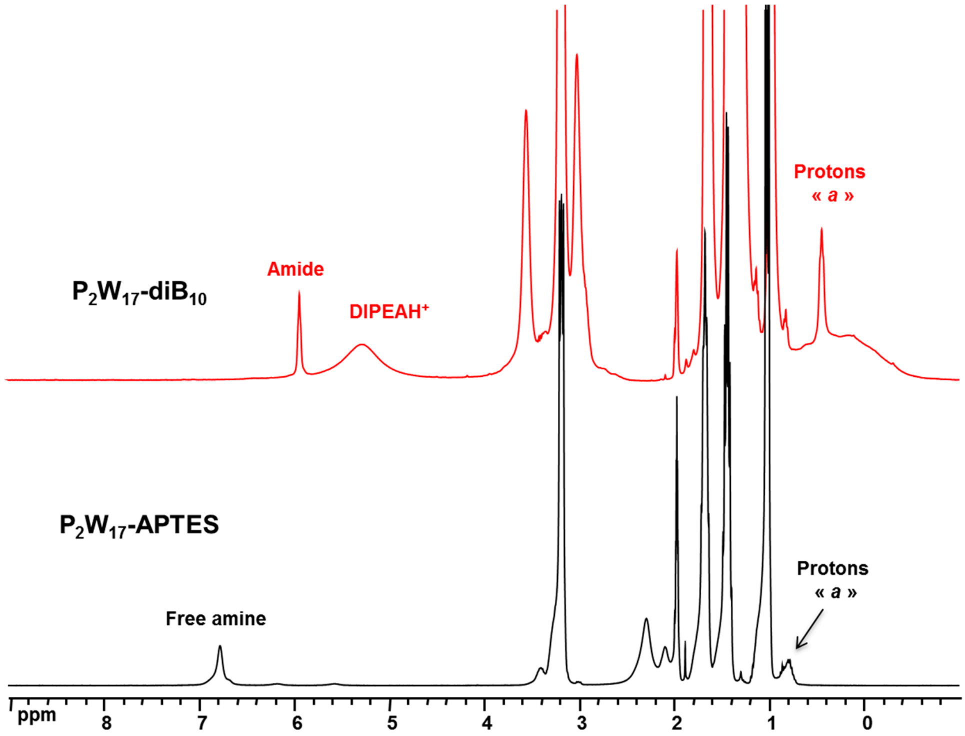


***Figure S27.*** *$^{1}$H NMR spectra of $P_2W_{17}$-di$B_{10}$ and $P_2W_{17}$-APTES in $CD_3CN$.*

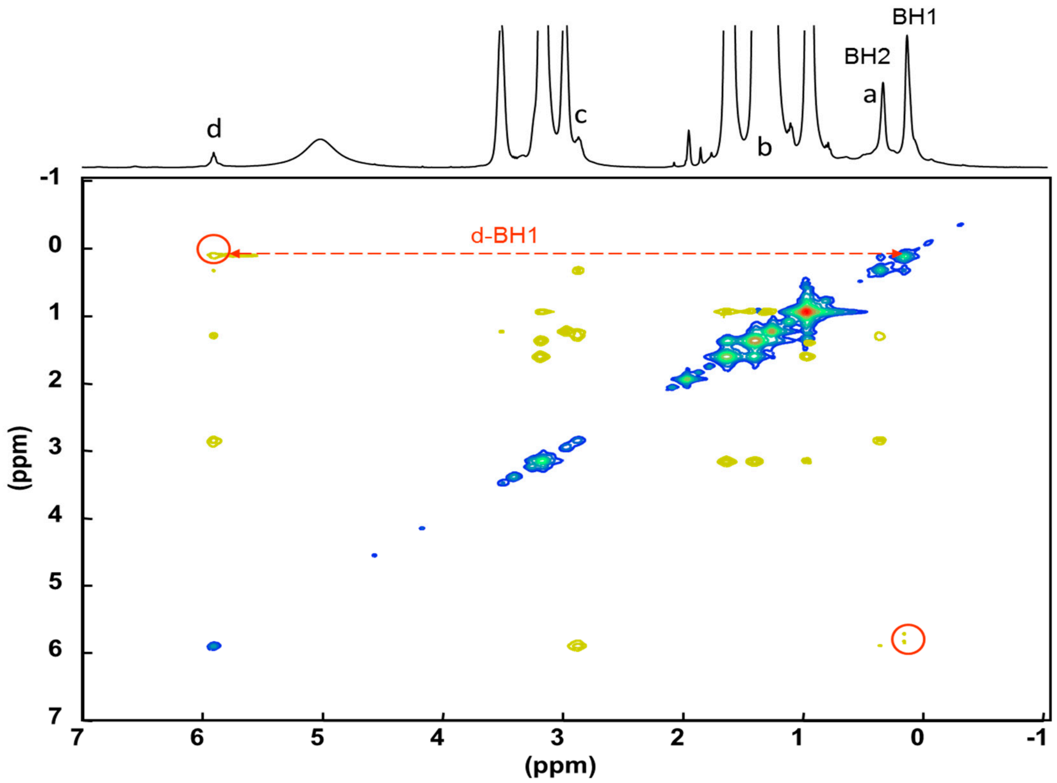


**Figure S28** $^{1}H\{^{11}B\}$ *ROESY NMR spectrum of* $SiW_{10}$*-di*$B_{10}$ *in* $CD_3CN$

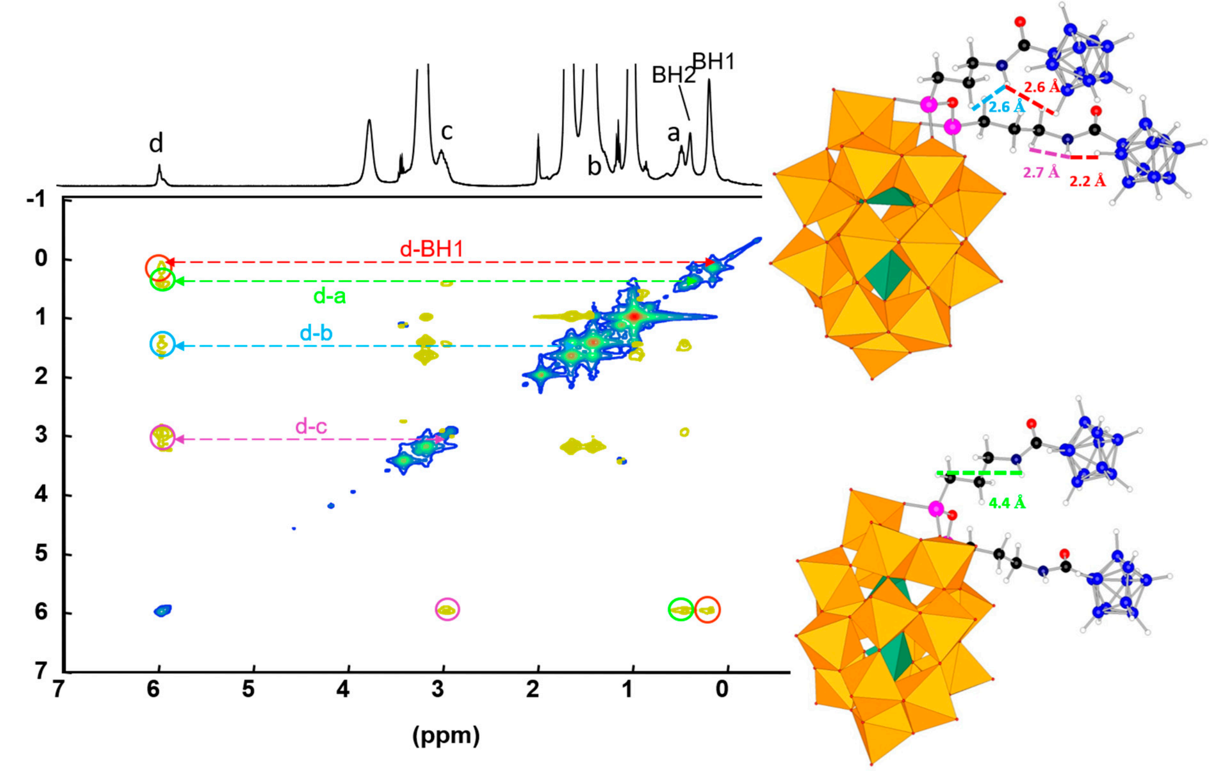


***Figure S29:*** $^{1}H\{^{11}B\}$ *ROESY NMR of* $P_2W_{17}$*-di*$B_{10}$ *in* $CD_3CN$*. The optimized structures are given for better understanding of the interaction evidenced by the experiments. The colors of dashed lines on the structures correspond to the colors of correlations evidenced on the 2D spectrum.*

### $^{13}$C NMR experiments.

The $^{13}C\{^1H\}$ NMR spectra of **$SiW_{10}$-APTES**, **$SiW_{10}$-monoB$_{10}$**, **$SiW_{10}$-diB$_{10}$, $P_2W_{17}$-APTES, and $P_2W_{17}$-diB$_{10}$** recorded in $CD_3CN$ are given in Figures S30 and S31 (SI). The signals of APTES carbon nuclei appeared split into two components in the mono-adduct compound **$SiW_{10}$-monoB$_{10}$** compared to the starting precursor **$SiW_{10}$-APTES** or the di-adduct product **$SiW_{10}$-diB$_{10}$** which both exhibit one set of three signals corresponding to the methylenic groups *a*, *b* and *c* of the APTES linker. Furthermore, a broad signal with quadruplet feature ($^1J$ coupling between $^{11}$B and $^{13}$C) at ca. 203 ppm can be also seen accounting for the carbonyl of the amide function attached to the Boron cluster.[20,21] Similar $^{13}C\{^1H\}$ NMR spectra -were observed for **$P_2W_{17}$-APTES** and **$P_2W_{17}$-diB$_{10}$** and this assignment is confirmed by 2D $^1$H-$^{13}$C HMBC experiment on **$SiW_{10}$-monoB$_{10}$** evidencing the correlation of these two signals (Figure S32, SI)

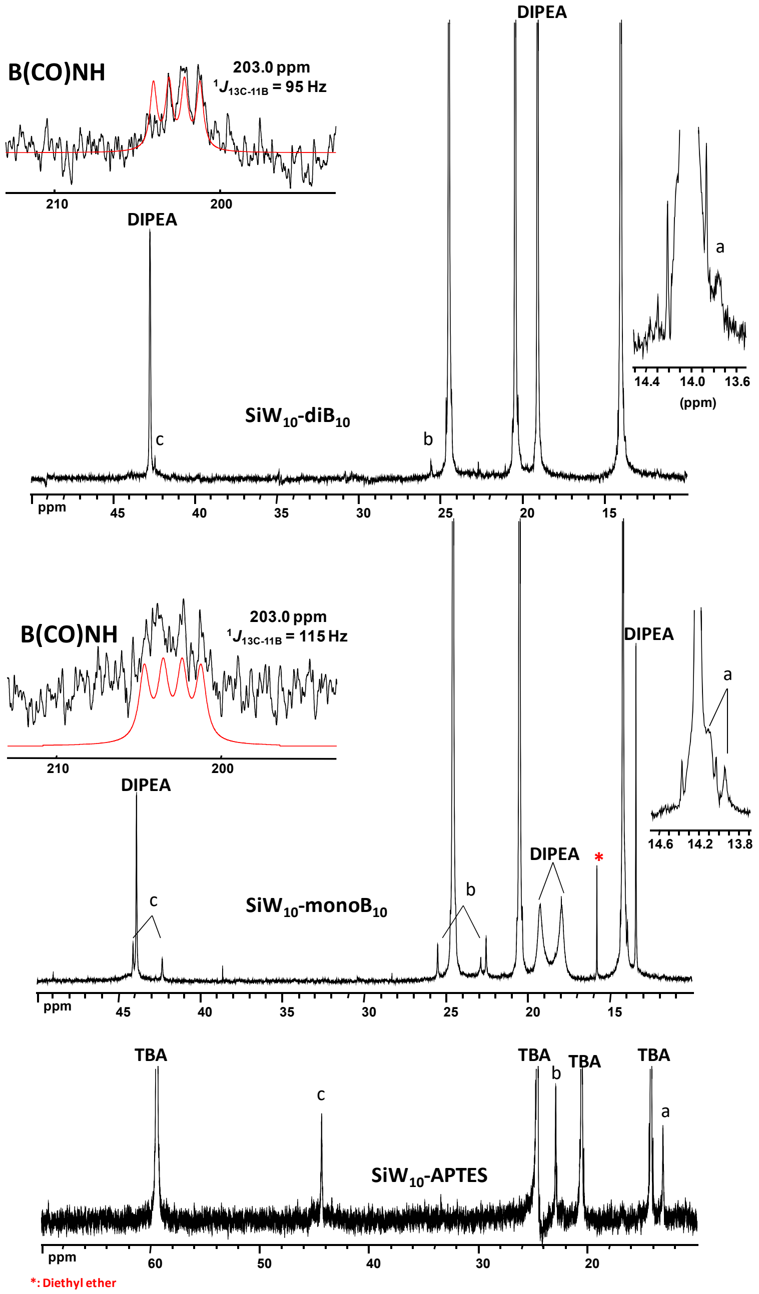


***Figure S30*** *$^{13}$C NMR spectra of $SiW_{10}$-monoB$_{10}$, $SiW_{10}$-diB$_{10}$ and $SiW_{10}$-APTES in $CD_3CN$*

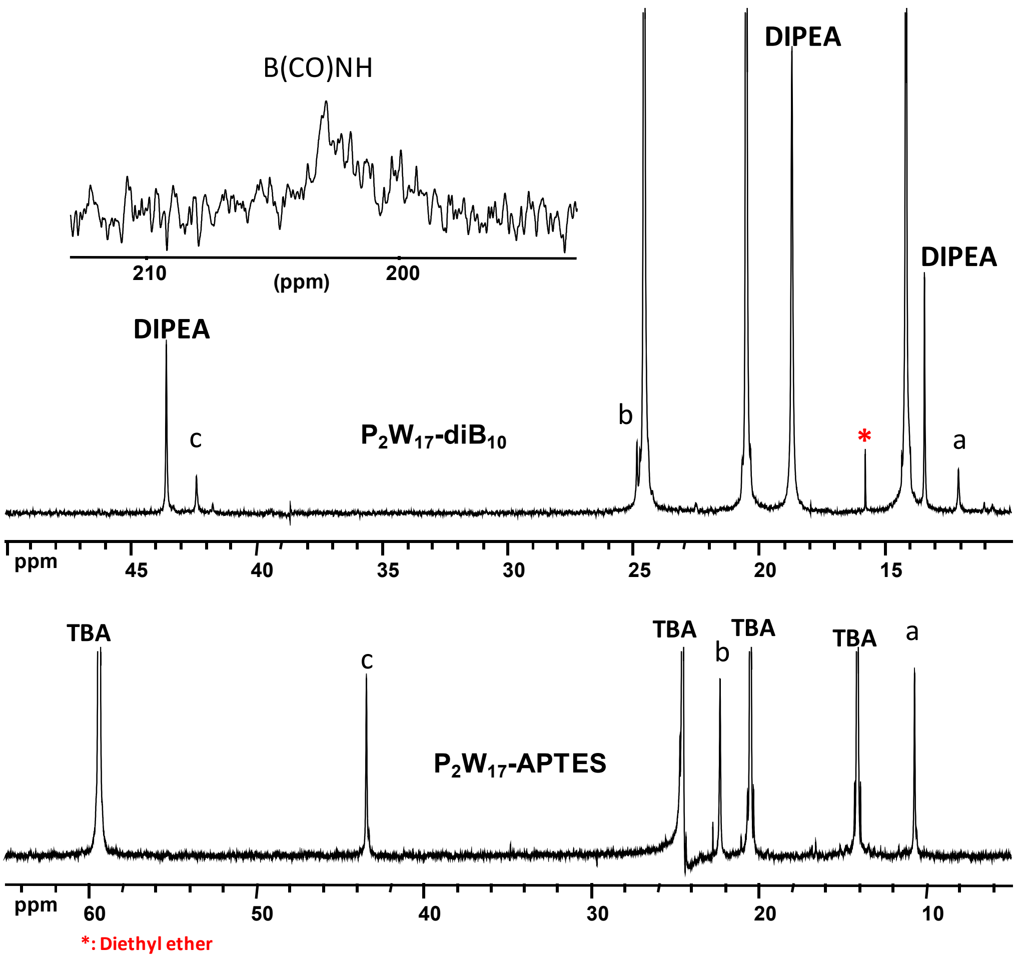


***Figure S31.*** *$^{13}$C NMR spectra of $P_2W_{17}$-diB$_{10}$ and $P_2W_{17}$-APTES in $CD_3CN$. The labels a, b and c correspond to the three methylenic carbon atoms of the APTES linker.*

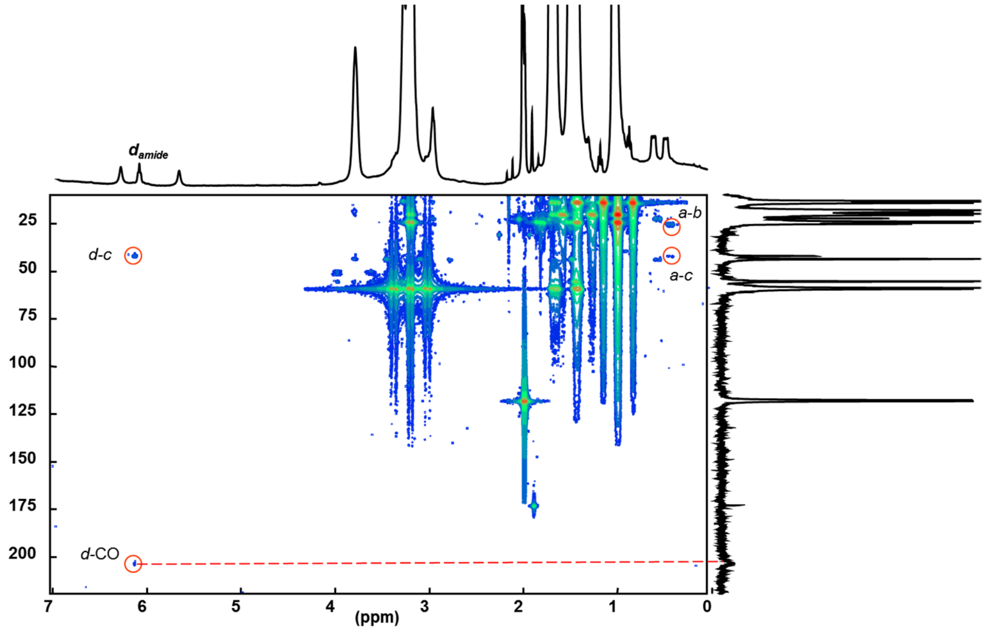


**Figure S32.** 2D $^1H$-$^{13}C$ *HMBC* NMR spectrum of $SiW_{10}$-monoB$_{10}$ in $CD_3CN$ highlighting the correlation between the signal assigned to the amide function with the C atom assigned to CO group of the adduct.

## Part 3 : DFT calculation

**Computational Details.** All results presented herein correspond to full geometry optimizations carried out by means of the Amsterdam Density Functional package ADF.[2] The GGA plus Grimme's empirical dispersion BP86-D functional[3] was used together with a Slater triple-ζ plus polarization basis set in all atoms. Relativistic corrections were introduced by the scalar-relativistic zero- order regular approximation (ZORA).[4] Solvent effects were included by using the continuum model COSMO.[5] For constructing the solvent cavity, the atomic radii values chosen were those Van der Waals radii from Klamt.[6] Vibrational harmonic frequencies were computed analytically, and entropic and thermal corrections were combined with the electronic energy to obtain the Gibbs free energy.

A data set collection of computational results is available in the ioChem-BD repository[7] and can be accessed via http://dx.doi.org/10.19061/iochem-bd-1-217.

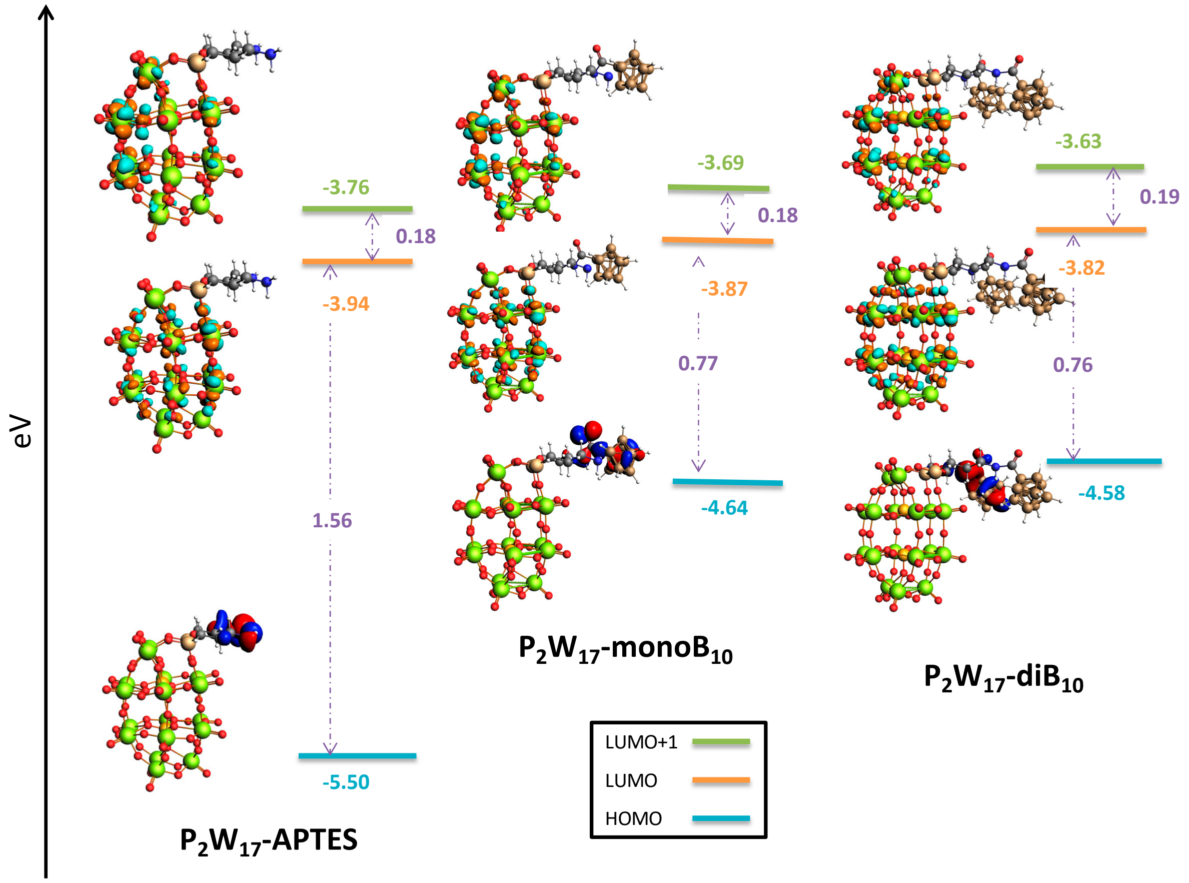


***Figure S33***. *Frontier orbitals energies (eV) for the* ***$P_2W_{17}$-APTES***, ***$P_2W_{17}$-monoB$_{10}$*** *and* ***$P_2W_{17}$-diB$_{10}$*** *species. Colour code: W green, O red, Si light brown, B dark brown, C grey, N blue, H white; HOMO: red/blue; LUMO: orange/cyan. MO surfaces plotted at a 0.03 isovalue.*

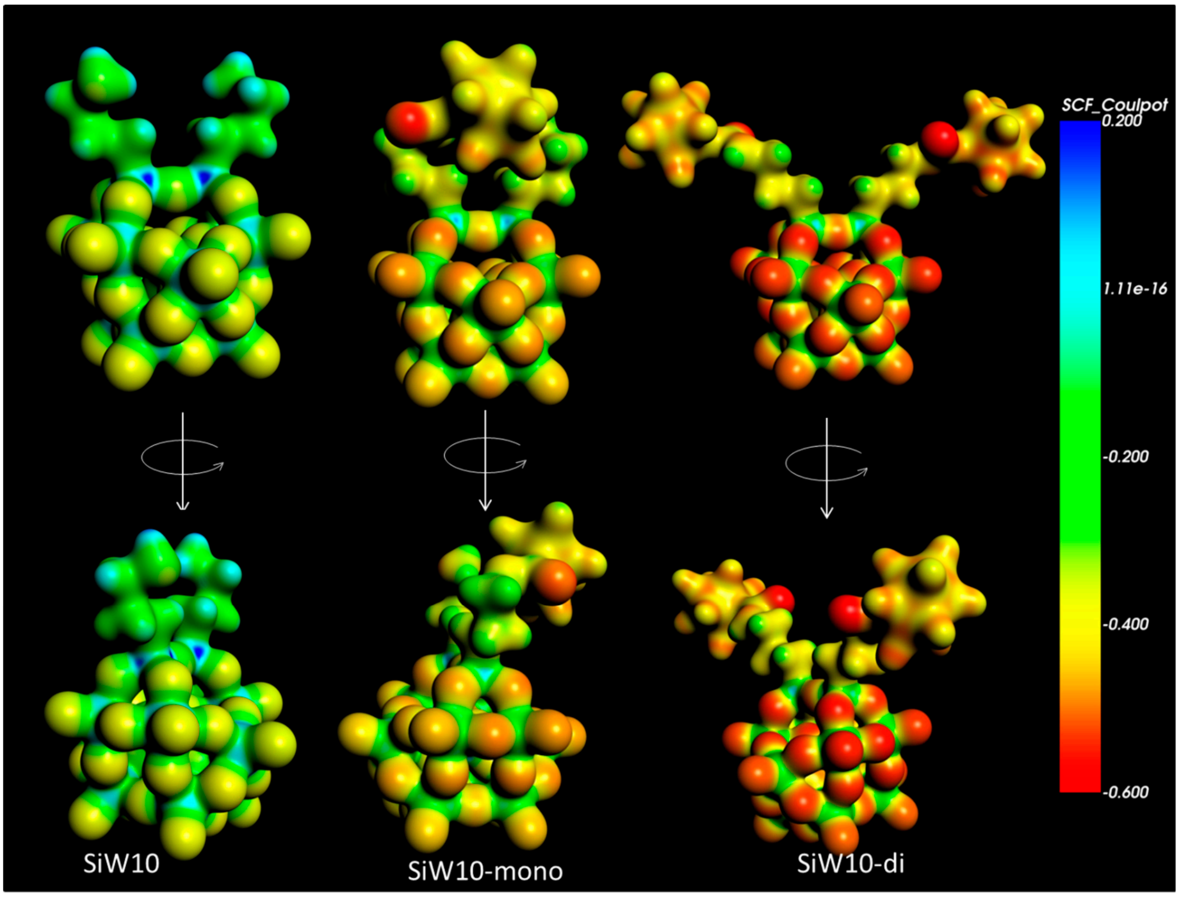


**Figure S34.** Two views of the molecular electrostatic potential in atomic units (a.u.) projected onto an electron density isosurface (0.03 e·au$^{-3}$) for the *SiW$_{10}$-APTES*, *SiW$_{10}$-monoB$_{10}$* and *SiW$_{10}$-diB$_{10}$* species.

# Part 4. Electronic and electrochemical studies

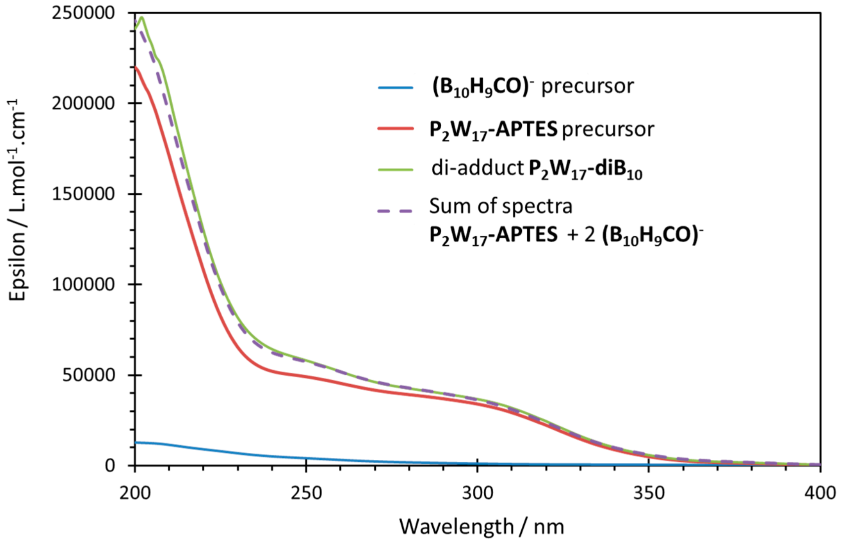


***Figure S35.*** *Electronic spectrum of $P_2W_{17}$-di$B_{10}$ (green line) in $CH_3CN$ containing 0.1 M $TBAClO_4$ at 20°C and C = 2.10$^{-4}$ mol.L$^{-1}$ in comparison with the spectra of the precursors $P_2W_{17}$-APTES (red line) and $[B_{10}H_9CO]^-$ (blue line). The sum of spectra of $P_2W_{17}$-APTES + 2 $[B_{10}H_9CO]^-$ is given in dashed violet line.*

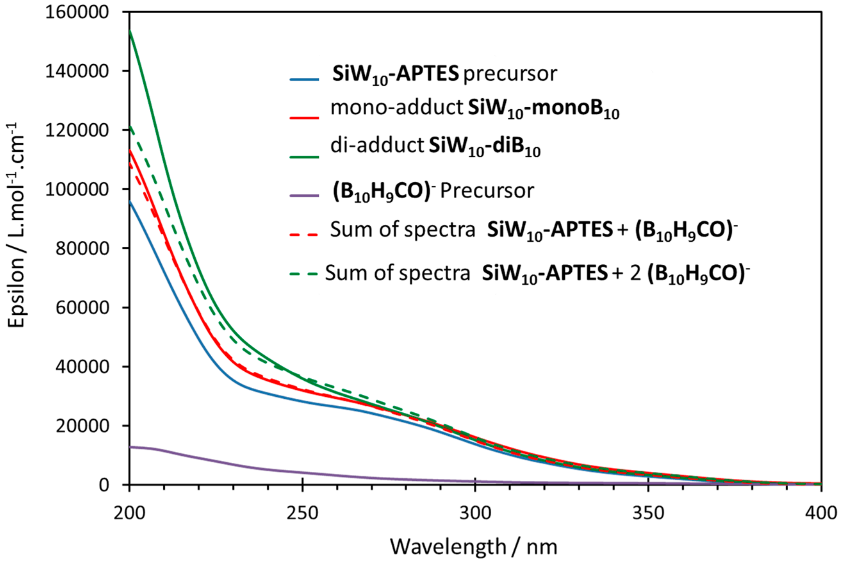


***Figure S36.*** *Electronic spectra of $SiW_{10}$-mono$B_{10}$ (red line) and $SiW_{10}$-di$B_{10}$ (green line) in $CH_3CN$ containing 0.1 M $TBAClO_4$ at 20°C and C = 2.10$^{-4}$ mol.L$^{-1}$ in comparison with the spectra of the precursors $SiW_{10}$-APTES (blue line) and $[B_{10}H_9CO]^-$ (violet line). The sum of spectra of $SiW_{10}$-APTES + 1 $[B_{10}H_9CO]^-$ is given in dashed red line while the sum of spectra of $SiW_{10}$-APTES + 2 $[B_{10}H_9CO]^-$ is given in dashed green line.*

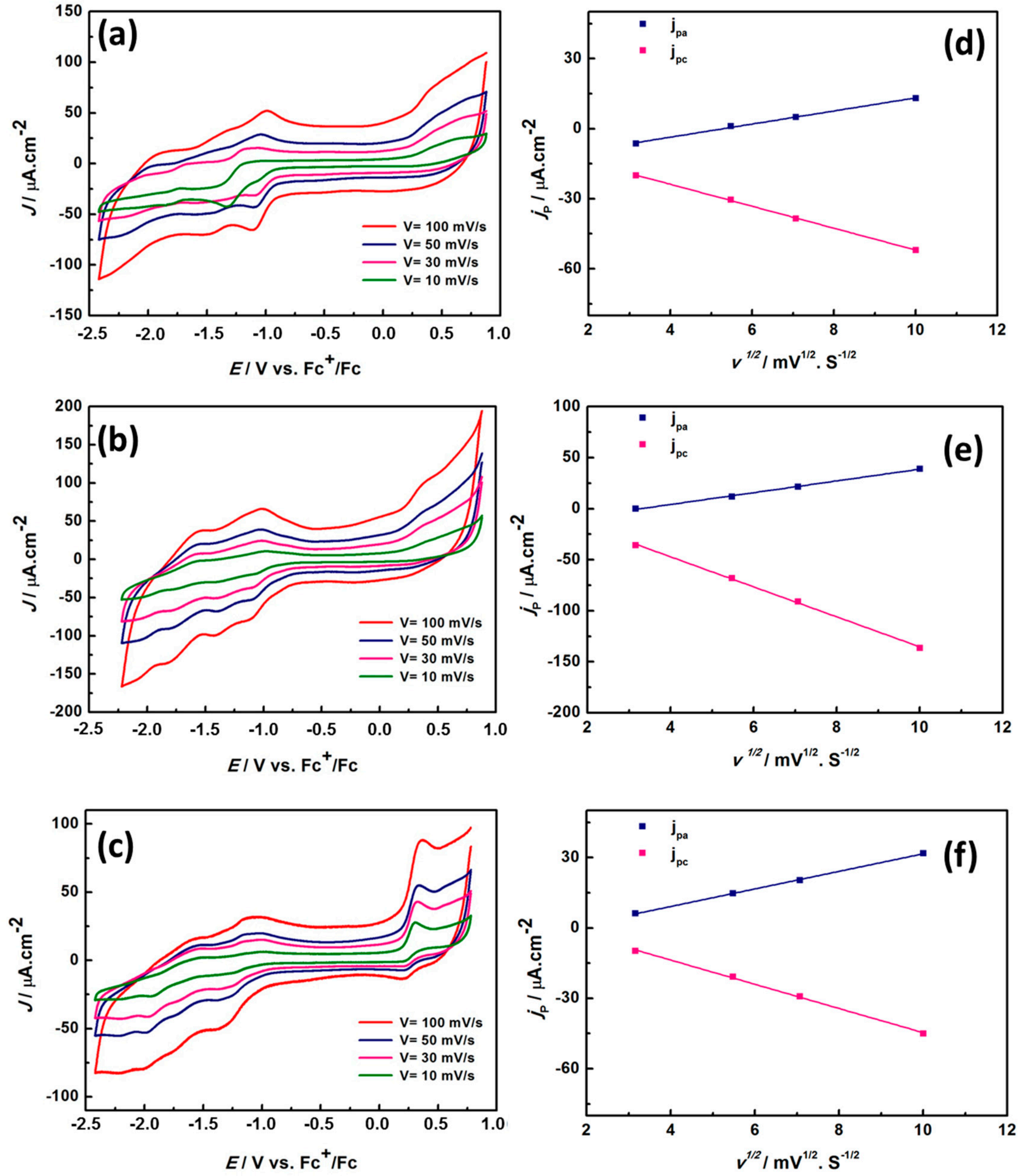


***Figure S37***. *Cyclic voltammograms (CVs) with different scan rates (from 10 to 100 mV $s^{-1}$) of 0.2 mM solution of $SiW_{10}$-APTES (a),* ***$SiW_{10}$-monoB$_{10}$*** *(b) and* ***$SiW_{10}$-diB$_{10}$*** *(c) in $CH_3CN$ + 0.1 M $TBAClO_4$. The reference electrode was a saturated calomel electrode (SCE). Figure (d), (e) and (f) represent the variation of the anodic and cathodic peak current density corresponding to the first wave as a function of the square root of the scan rate, respectively for $SiW_{10}$-APTES,* ***$SiW_{10}$-monoB$_{10}$*** *and* ***$SiW_{10}$-di10.*** *The variations of the cathodic and anodic currents associated with the first reduction waves appear linear as a function of the square root of the scan rate which demonstrates that the electrochemical processes of these species are controlled by diffusion.*

.

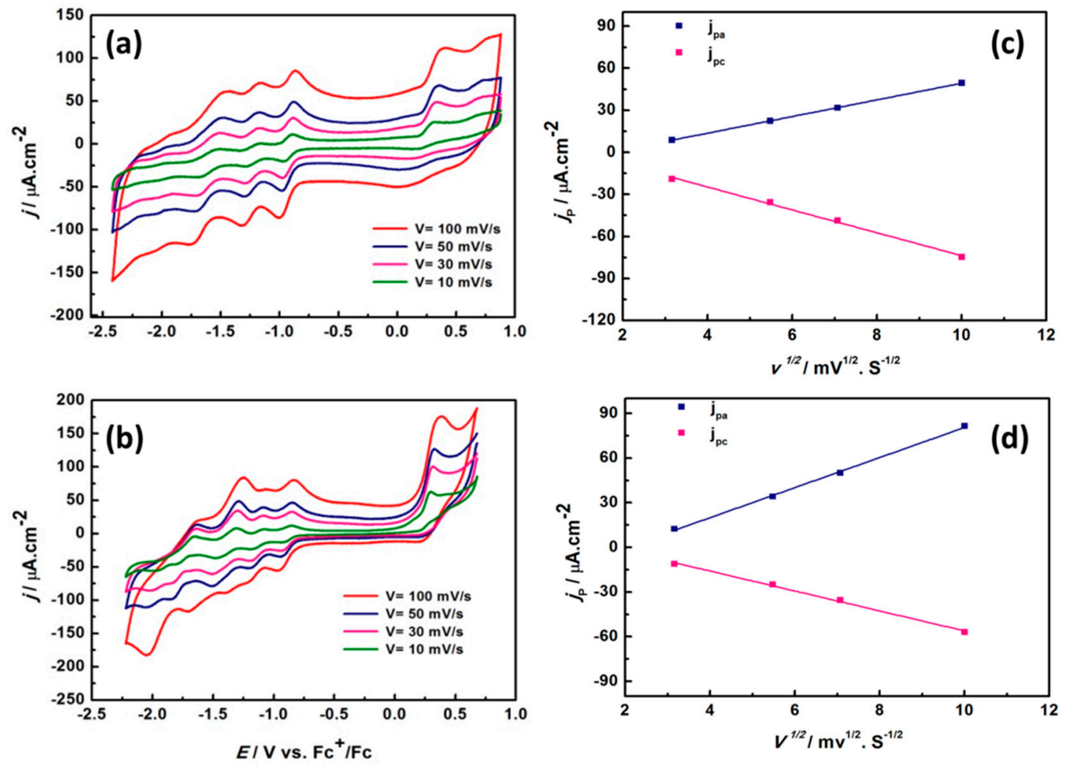


***Figure S38.*** *Cyclic voltammograms (CVs) of 0.2 mM solution of for $P_2W_{17}$-APTES (a) and $P_2W_{17}$-di$B_{10}$ (b) in $CH_3CN$ + 0.1 M $TBAClO_4$ with different scan rates (from 10 to 100 mV $s^{-1}$). Figures (c) and (d) represent the variations of the anodic and cathodic peak current density associated with the first reduction wave of $P_2W_{17}$-APTES (c) and $P_2W_{17}$-di10 (d) as a function of the square root of the scan rate. The variations of the cathodic and anodic currents associated with the first reduction waves appear linear as a function of the square root of the scan rate which demonstrates that the electrochemical processes of these species are controlled by diffusion.*

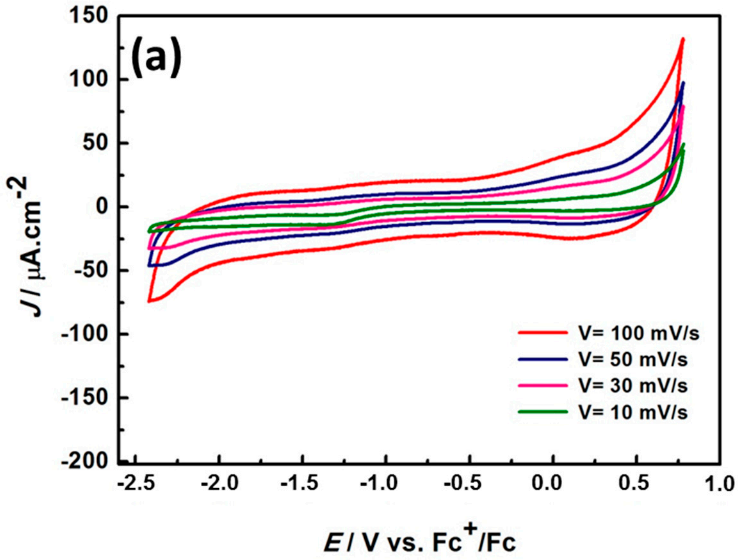


***Figure S39.*** *(a) Cyclic voltammograms (CVs) of TBA[$B_{10}H_9CO$] cluster of concentration of $2.10^{-4}$ M. The electrolyte was $CH_3CN$ + 0.1 M $TBAClO_4$*

## Electrocatalytic properties for the reduction of protons into hydrogen (H.E.R.)

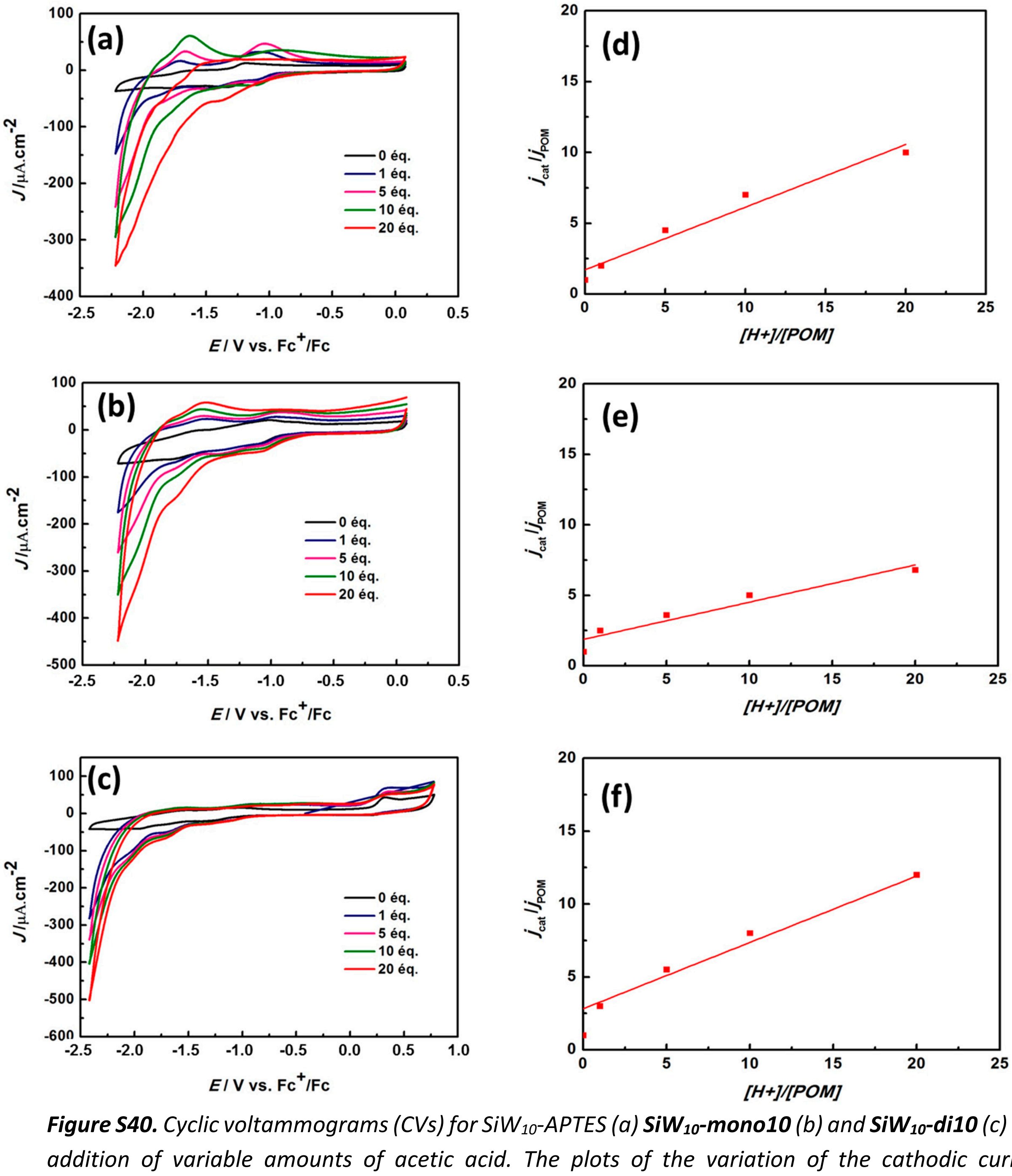


***Figure S40.*** *Cyclic voltammograms (CVs) for* $SiW_{10}$*-APTES (a)* ***$SiW_{10}$-mono10*** *(b) and* ***$SiW_{10}$-di10*** *(c) after addition of variable amounts of acetic acid. The plots of the variation of the cathodic currents measured at -2.2 V vs Fc+/Fc as a function of the ratio [acid]/[POM] for* $SiW_{10}$*-APTES,* ***$SiW_{10}$-mono10*** *and* ***$SiW_{10}$-di10*** *are given in (d), (e) and (f) respectively. In all cases, the electrolyte was* $CH_3CN$ *+ 0.1M* $TBAClO_4$*.*

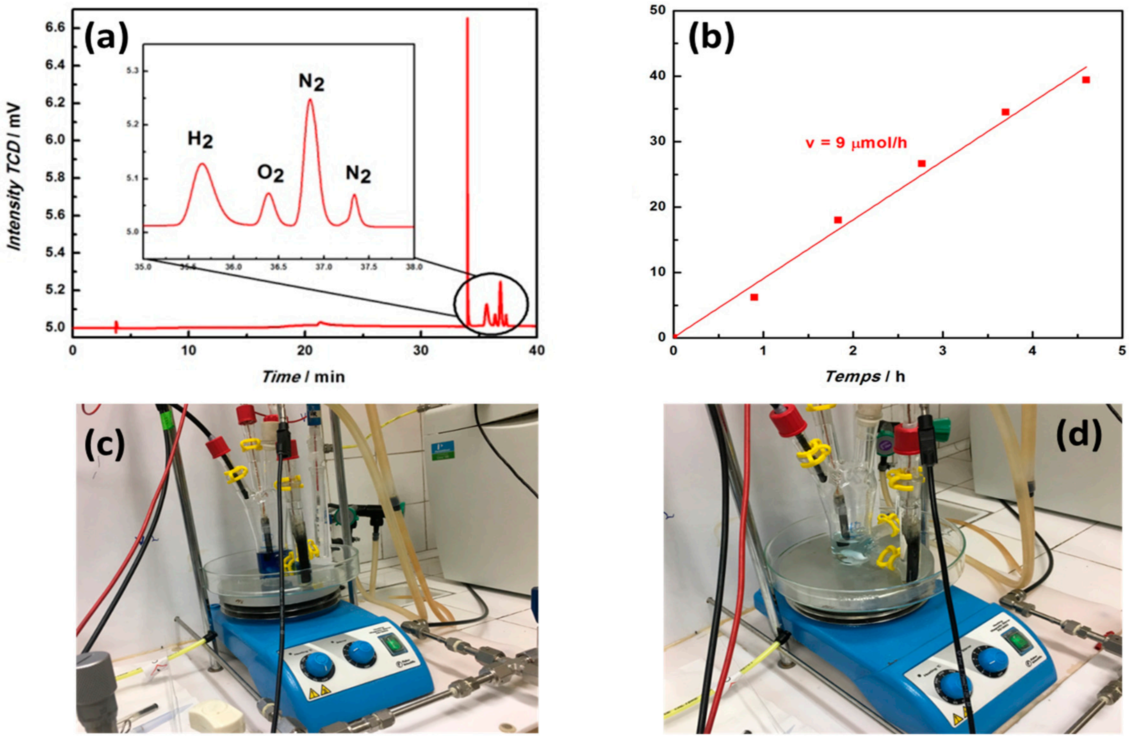


***Figure S41.*** (a) Gas chromatograph of the gas phase analysed during the electrolysis of $P_2W_{17}$-di$B_{10}$; (b) rate of hydrogen evolution measured during 4.5 h of the electrolysis of $P_2W_{17}$-di$B_{10}$; (c) picture of the electrolysis cell during the electrolysis. The blue color is typical of reduced POM; (d) picture of the cell after the electrolysis. The POM comes back to its initial color, indicating that the reduced POM is the active species for the reduction of protons

**Table S3. Electrochemical data.**

| Compound | Anodic and Cathodic potentials, $E_{pa}$ and $E_{pc}$, observed for reduction waves. $E_{pa}$ and $E_{pc}$ are given *vs.* $Fc^+/Fc$ |
|---|---|
| **$SiW_{10}$-APTES** | $E_{pc}$ = -1.116 V, $E_{pc}$ = -1.565 V, $E_{pc}$ = -1.950 V |
| | $E_{pa}$ = -1.137 V, $E_{pa}$ = -1.336 V, $E_{pa}$ = -2.200 V |
| **$SiW_{10}$-mono$B_{10}$** | $E_{pc}$ = -1.124 V, $E_{pc}$ = -1.415 V, $E_{pc}$ = -1.819 V |
| | $E_{pa}$ = -1.150 V, $E_{pa}$ = -1.210 V, $E_{pa}$ = -1.650 V |
| **$SiW_{10}$-di$B_{10}$** | $E_{pc}$ = -1.250 V, $E_{pc}$ = -1.750 V, $E_{pc}$ = -2.150 V |
| | $E_{pa}$ = -1.120 V, $E_{pa}$ =-1.610 V, $E_{pa}$ = -1.910 V |
| **$P_2W_{17}$-APTES** | $E_{pc}$ = -1.020 V, $E_{pc}$ = -1.317 V, $E_{pc}$ = -1.715 V, $E_{pc}$ = -2.070 V |
| | $E_{pa}$ = -0.860 V, $E_{pa}$ = -1.150 V, $E_{pa}$ = -1.450 V, $E_{pa}$ = -1.920 V |
| **$P_2W_{17}$-di$B_{10}$** | $E_{pc}$ = -0.955 V, $E_{pc}$ = -1.180 V, $E_{pc}$ = -1.390 V, $E_{pc}$ = -1.790 V |
| | $E_{pa}$ = -0.830 V, $E_{pa}$ = -1.100 V, $E_{pa}$ = -1.250 V, $E_{pa}$ = -1.630 V |

## References of the supporting information


1. Robin K. Harris, E. D. B., Sonia M. Cabral De Menezes, Robin Goodfellow, Pierre Granger, NMR Nomenclature. Nuclear Spin Properties and Conventions for Chemical Shifts. *Pure Appl. Chem.* **2001,** *73*, 1795–1818.

2. a) G. te Velde, F. M. Bickelhaupt, E. J. Baerends, C. F. Guerra, S. J. A. Van Gisbergen, J. G. Snijders and T. Ziegler, *Journal of Computational Chemistry* **2001**, *22*, 931-967; b) C. F. Guerra, J. G. Snijders, G. te Velde and E. J. Baerends, *Theor. Chem. Acc.* **1998**, *99*, 391-403; c) E. Vanlenthe, E. J. Baerends and J. G. Snijders, *Journal of Chemical Physics* **1994**, *101*, 9783-9792.

3. a) A. D. Becke, *Journal of Chemical Physics* **1993**, *98*, 5648-5652; b) J. P. Perdew, *Physical Review B* **1986**,*33*,8822-8824.

4. a) E. van Lenthe, A. Ehlers and E. J. Baerends, *Journal of Chemical Physics* **1999**, *110*, 8943-8953; b) E. Vanlenthe, E. J. Baerends and J. G. Snijders, *Journal of Chemical Physics* **1993**, *99*, 4597-4610.

5. a) C. C. Pye and T. Ziegler, *Theor. Chem. Acc.* **1999**, *101*, 396-408; b) A. Klamt and G. Schuurmann, *Journal of the Chemical Society-Perkin Transactions 2* **1993**, 799-805.

6. A. Klamt, V. Jonas, T. Burger and J. C. W. Lohrenz, *Journal of Physical Chemistry A* **1998**, *102*, 5074-5085.

7. M. Alvarez-Moreno, C. de Graaf, N. Lopez, F. Maseras, J. M. Poblet and C. Bo, *Journal of Chemical Information and Modeling* **2015**, *55*, 95-103.

8. Bonchio, M.; Carraro, M.; Scorrano, G.; Bagno, A., Photooxidation in water by new hybrid molecular photocatalysts integrating an organic sensitizer with a polyoxometalate core. *Adv. Synth. Catal.* **2004,** *346* (6), 648-654.

9. Berardi, S.; Carraro, M.; Iglesias, M.; Sartorel, A.; Scorrano, G.; Albrecht, M.; Bonchio, M., Polyoxometalate-Based N-Heterocyclic Carbene (NHC) Complexes for Palladium-Mediated C-C Coupling and Chloroaryl Dehalogenation Catalysis. *Chem.-Eur. J.* **2010,** *16* (35), 10662-10666.

10. Modugno, G. M., Angèle; Bonchio, Marcella; Albrecht, Martin; Carraro, Mauro, Transfer Hydrogenation Catalysis by a N-Heterocyclic Carbene (NHC) Iridium Complex on a Polyoxometalate Platform. *Eur. J. Inorg. Chem.* **2014,** *14*, 2356-2360.

11. Carraro, M.; Modugno, G.; Fiorani, G.; Maccato, C.; Sartorel, A.; Bonchio, M., Organic-Inorganic Molecular Nano-Sensors: A Bis-Dansylated Tweezer-Like Fluoroionophore Integrating a Polyoxometalate Core. *Eur. J. Org. Chem.* **2012,** (2), 281-289.

12. Cedric R. Mayer, S. N., and Valerie Cabuil, A Nanoscale Hybrid System Based on Gold Nanoparticles and Heteropolyanions. *Angew. Chem.-Int. Edit.* **2002,** *41*.

13. Izzet, G.; Volatron, F.; Proust, A., Tailor-made Covalent Organic-Inorganic Polyoxometalate Hybrids: Versatile Platforms for the Elaboration of Functional Molecular Architectures. *Chem Rec* **2017,** *17* (2), 250-266.

14. Abi-Ghaida, F.; Laila, Z.; Ibrahim, G.; Naoufal, D.; Mehdi, A., New triethoxysilylated 10-vertex closo-decaborate clusters. Synthesis and controlled immobilization into mesoporous silica. *Dalton Trans* **2014,** *43* (34), 13087-95.

15. Mayer, C. R.; Roch-Marchal, C.; Lavanant, H.; Thouvenot, R.; Sellier, N.; Blais, J. C.; Secheresse, F., New organosilyl derivatives of the Dawson polyoxometalate$[\alpha_2\text{-}P_2W_{17}O_{61}(RSi)_2O]^{6-}$: Synthesis and mass spectrometric investigation. *Chem.-Eur. J.* **2004,** *10* (21), 5517-5523.

16. Inorganic Synthesis. *Wiley-Interscience publication* **1990,** *27*.

17. Mbomekalle, I. M.; Lu, Y. W.; Keita, B.; Nadjo, L., Simple, high yield and reagent-saving synthesis of pure alpha-$K_6P_2W_{18}O_{62}.14H_2O$. *Inorg. Chem. Commun.* **2004,** *7* (1), 86-90.

18. Kenneth Shelly, C. B. K., and M. Frederick Hawthorne, Synthesis of Monosubstituted Derivatives of closo -Decahydrodecaborate( 2-) . X-ray Crystal Structures of [closo-2-$B_{10}H_9CO]^-$ and [closo-2-$B_{10}H_9NCO]^{2-}$. *Inorg. Chem.* **1992,** *31*, 2889-2892.

19. Mayer, C. R. F., I.; Thouvenot, R., Bis- and tetrakis(organosilyl) decatungstosilicate, [ -$SiW_{10}O_{36}(RSi)_2O]^{4-}$ and [$\gamma$- $SiW_{10}O_{36}(RSiO)_4]^{4-}$: Synthesis and structural determination by multinuclear NMR spectroscopy and matrix-assisted laser desorption/ionization time-of-flight mass spectrometry. *Chemistry – A European Journal* **2000,** *6* (1), 105-110.

20. Wrackmeyer, B.; Hernandez, Z. G.; Lang, J.; Tok, O. L. *Anorg. Z.,* 1,2-, 1,7- and 1,12-Dicarba-*closo*-dodecaborane(12) Derivatives Revisited by $^{13}$C NMR Spectroscopy and DFT Calculations. First Observation of Isotope-Induced Chemical Shifts $^1\Delta^{10/11}B(^{13}C)$, and the Signs and Magnitudes of Coupling Constants $^1J(^{13}C,^{13}C)$ and $^1J(^{13}C,^{11}B)$. *Allg. Chem.* 2009, *635*, 1087-1093.

21. Wrackmeyer, B.; Long-range nuclear spin-spin coupling between $^{11}$B and $^{13}$C, $^{29}$Si or $^{119}$Sn: a promising tool for structural assignment. *Polyhedron* 1986, *5*, 1709-1721.
